# Time-restricted feeding extends healthspan in both sexes and lifespan in male C57BL/6 J mice

**DOI:** 10.1038/s43587-026-01129-8

**Published:** 2026-06-02

**Authors:** Samantha E. Iiams, Nathan J. Skinner, Mary Wight-Carter, Victoria A. Acosta-Rodríguez, Carla B. Green, Joseph S. Takahashi

**Affiliations:** 1 Department of Neuroscience, Peter O’Donnell Jr. Brain Institute, University of Texas Southwestern Medical Center, Dallas, TX USA; 2 Animal Resource Center, University of Texas Southwestern Medical Center, Dallas, TX USA; 3 Howard Hughes Medical Institute, University of Texas Southwestern Medical Center, Dallas, TX USA; 4 Present Address: Circadian Biology of Aging Unit, Translational Gerontology Branch, National Institute on Aging (NIA/NIH), Baltimore, MD USA

**Keywords:** Ageing, Circadian rhythms and sleep, Metabolism

## Abstract

Time-restricted feeding (TRF) aligned with an organism’s circadian rhythm has been shown to improve health, but its long-term effects on healthspan and lifespan in mammals, especially under standard dietary conditions that do not promote obesity, remain unclear. Here, we examined the impact of 12-h and 8-h nightly TRF windows in 264 male and 264 female C57BL/6 J mice fed regular chow. TRF improved multiple health measures, including behavioral rhythmicity, body weight and composition, frailty, and disease onset. These effects were most pronounced in the 8-h TRF group, which exhibited voluntary caloric restriction in addition to time restriction. A composite Healthspan Index revealed that TRF extended healthspan in both sexes, though the benefits were more prolonged in female mice relative to their total lifespan. Median lifespan was significantly extended in male mice under 8-h TRF by 12%, whereas female mice showed no significant lifespan extension. These results demonstrate sex-specific effects of TRF on mammalian aging.

## Abstract (web summary)

Assessing over 500 male and female C57BL/6 J mice fed a standard diet, Iiams, Skinner and colleagues report that early-onset lifelong time-restricted feeding improved healthspan in both sexes but extended median lifespan only in male mice, revealing sex-specific effects on mammalian aging.

## Main

Although human life expectancy has nearly doubled since the early 20th century^1^, healthspan remains limited, with many spending over 10% of their lives in poor health due to age-related functional declines and disease development^2^. Caloric restriction (CR), a potent aging intervention involving a 30–50% reduction in food intake without malnutrition, has been extensively studied for its ability to delay the onset of age-associated diseases, including cancer, cardiovascular disorders and neurodegeneration, while extending lifespan across multiple model organisms^3–5^. Incorporating daily feeding/fasting cycles with CR enhances these geroprotective effects^6–9^, and aligning feeding with an organism’s active circadian phase can further extend lifespan^10^. This synergy likely reflects both the synchronization of nutrient intake with internal metabolic processes and the protective effects of temporal compartmentalization, driven by circadian clock-regulated gene expression in the liver and other peripheral tissues^11–17^.

Aging triggers a progressive damping of circadian rhythmicity in both humans and animal models. This decline, evident in behavior and physiology, stems from a reduction in amplitude and shifted phases of the molecular clock’s transcriptional output^18^. Interventions that restore circadian rhythms can improve health and extend longevity in model organisms^19^, suggesting that optimizing meal timing is a promising strategy to counteract age-related rhythm disruptions and promote healthy aging^20^.

Given that CR is challenging to maintain in people^21^, meal timing alone via time-restricted feeding (TRF) in animals, or time-restricted eating (TRE) in humans, has emerged as an alternative strategy to delay aging^22,23^. TRF, which restricts feeding to specific daily windows without limiting overall caloric intake, can confer similar benefits as timed CR, including improved rhythmic gene expression, cognitive function and cardiometabolic health, even in obesity models fed a high-fat, high-sucrose diet^24–30^. Recent clinical TRE trials report high adherence and improved cardiometabolic outcomes in subjects with pre-existing conditions, demonstrating its potential as a feasible intervention to enhance human healthspan^31–35^.

However, few studies have examined the benefits of TRF/TRE under typical metabolic conditions without diet- or genetically induced obesity^36–38^, and even fewer have compared impacts between the sexes^39^. The long-term impact of TRF on healthspan and lifespan in mammalian models also remains unclear^40,41^. In this study, we sought to address these key knowledge gaps and determine whether TRF could serve as an effective aging intervention for healthy male and female mice.

## Results

### Testing early-onset circadian-aligned TRF

**Fig. 1:**
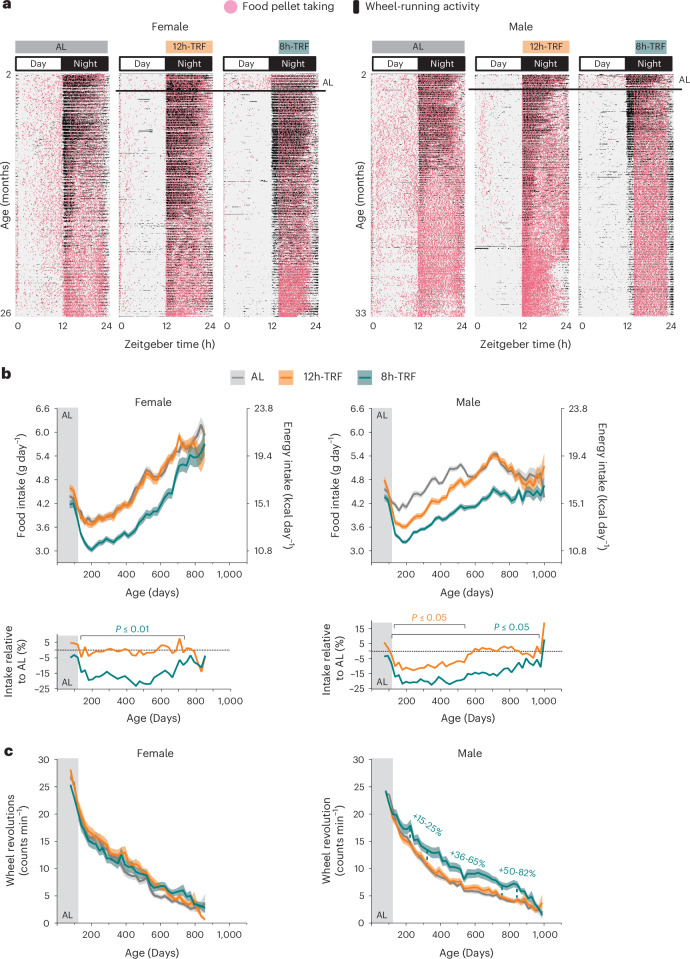
Circadian-aligned TRF reduces feeding and improves wheel-running activity in a feeding window- and sex-dependent manner. **a**, In female mice 2–26 months of age and male mice 2–33 months of age, representative actograms of individual mice show wheel-running (black mark) and food pellet taking (pink dot) activity over the course of the 24-h light:dark cycle. Black horizontal line shows ends of AL baseline feeding for the TRF groups up to 4 months of age. **b**, Longitudinal profiles of average daily food intake and energy intake with age (top) and percent differences in CR relative to AL controls with age (bottom). **c**, Longitudinal profiles of average daily wheel revolutions with age. In **b** and **c**, AL baseline feeding (gray area) and TRF feeding conditions (white area) are shown. Each point represents mean of 21 days ± standard error of the mean (s.e.m.) (shaded regions). Age blocks with significant differences (*P* ≤ 0.05) in TRF versus AL (colored *P* values: orange, 12-h TRF; teal, 8-h TRF) marked by brackets/percentages as determined by two-way analysis of variance (ANOVA) and Tukey’s post-hoc (full details of ANOVA results for each figure can be found in Supplementary Table 1). Starting number of mice for feeding/wheel profiles: female: AL, *n* = 103/96; 12-h TRF, *n* = 76/73; 8-h TRF, *n* = 76/71; male: AL, *n* = 97/96; 12-h TRF, *n* = 72/72; 8-h TRF, *n* = 71/73.

**Extended Data Fig. 1:**
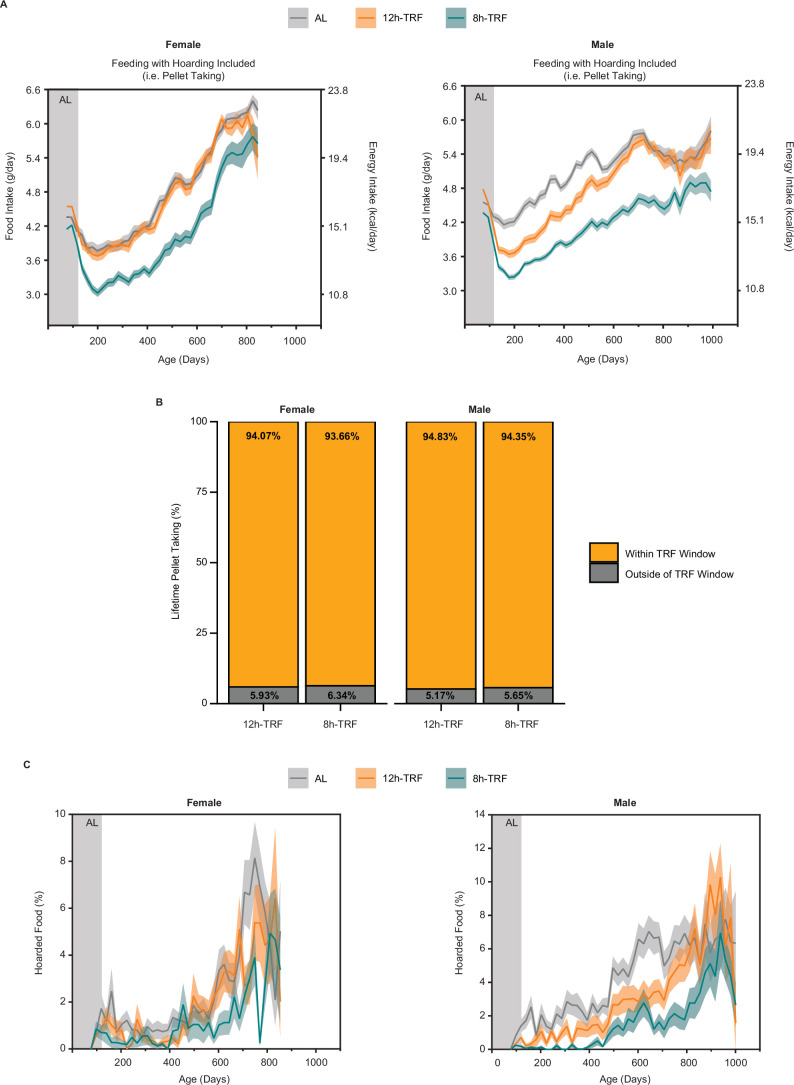
Food taking and hoarding behavior. 300 mg pellets are dispensed from the feeder one at a time for the mouse to retrieve. In female and male mice: (**a**) Longitudinal profiles of daily food taken from the feeder including uneaten (hoarded) food found in the cage (g/day) and the equivalent energy intake (kcal/day). (**b**) Lifetime averages of the percent (%) of food taken from the feeder within or outside of the TRF window. (**c**) Longitudinal profiles of hoarded food removed from cages as a % of the total food taken from the feeder. Panels A and C: *Ad libitum* (AL) baseline feeding (Gray area). Time-restricted feeding (TRF) (White area). Mean of 21 days ± SEM (shaded regions). All panels: Starting N for Feeding profiles: Female: AL, N=103. 12-h TRF, N=76. 8-h TRF, N=76. Male: AL, N=97. 12-h TRF, N=72. 8-h TRF, N=71.

**Extended Data Fig. 2:**
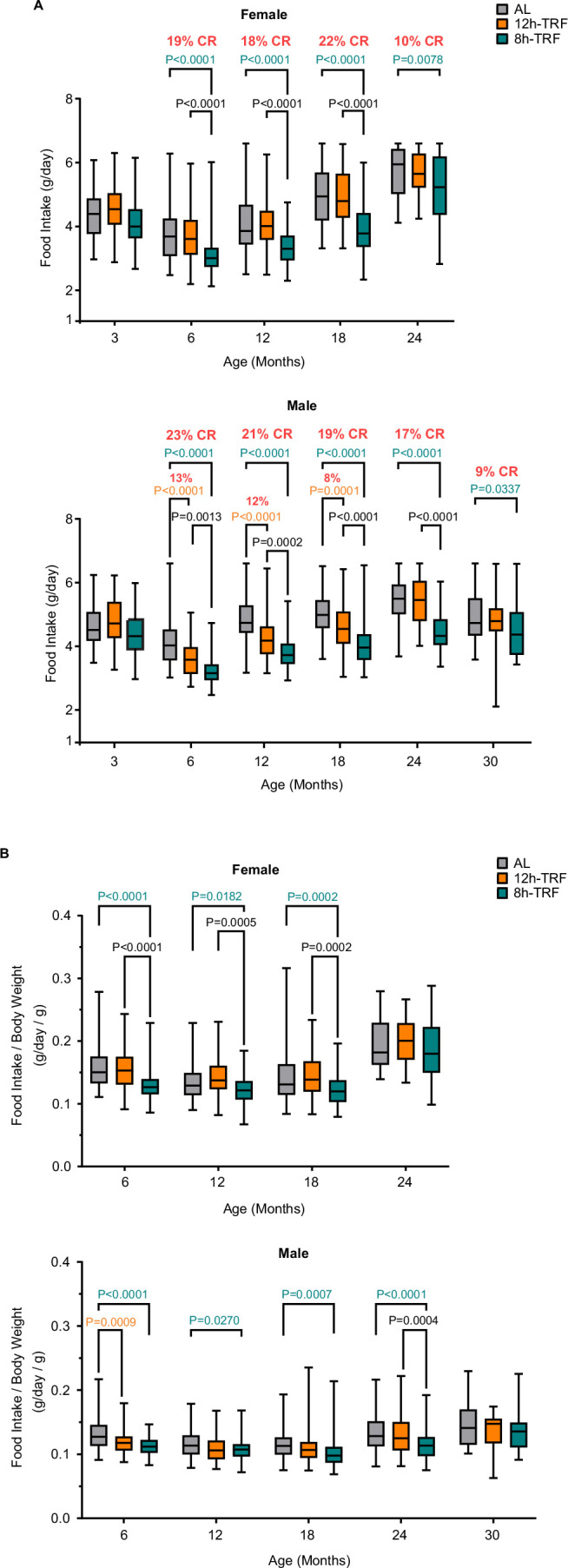
Food intake by monthly age epochs. (**a**) In female and male mice, box plots of food intake with hoarding removed (g/day) at 3 and 6 months of age and then every 6 months following. Food intake per mouse averaged every 21 days. Any caloric restriction (CR) relative to the AL control at each age epoch shown as %. Female 3, 6, 12, 18, and 24 months: AL, N=103, 101, 95, 81, 40. 12-h TRF, N=76, 74, 68, 58, 31. 8-h TRF, N=76, 74, 68, 61, 37. Male 3, 6, 12, 18, 24, and 30 months: AL, N=97, 96, 98, 96, 76, 23. 12-h TRF, N=72, 74, 72, 71, 61, 18. 8-h TRF, N=71, 74, 72, 72, 67, 35. (**b**) In female and male mice, boxplots of food intake (g/day) with hoarding removed relative to body weight (g) at 6 months of age and then every 6 months following. Female 6, 12, 18, and 24 months: AL, N=98, 91, 78, 35. 12-h TRF, N=72, 67, 57, 30. 8-h TRF, N=72, 67, 60, 36. Male 6, 12, 18, 24, and 30 months: AL, N=94, 93, 95, 76, 23. 12-h TRF, N=74, 73, 70, 60, 17. 8-h TRF, N=71, 71, 71, 67, 34. All panels: Box plots indicate the median (center line within box), interquartile range (25th–75th percentiles/bottom-top lines of box), and minimum and maximum values (whiskers). Significant differences (P≤0.05) in TRF vs AL (colored P-values; orange:12-h TRF, teal:8-h TRF) and 12-h vs 8-h TRF (black colored P-values) as determined by two-way ANOVA and Tukey’s post-hoc.

To determine how TRF impacts healthspan and lifespan in mammals under normal (non-obesogenic) nutritional conditions, we individually housed 264 female and 264 male C57BL/6 J mice in wheel cages with automated feeders beginning at 2 months of age and fed them purified precision pellets (Bio-Serv F0075). For the first 8 weeks, all mice were fed ad libitum (AL; maximum 22 pellets, 6.6 g, or 23.8 kcal/day). At 4 months of age, 78 mice/sex were assigned to a 12-h TRF feeding window (ZT12-24), 78 mice/sex to an 8-h TRF window (ZT14-22), and the remaining 108 mice/sex remained AL as controls for life. Both TRF regimens were circadian aligned to allow feeding during the nocturnal active phase. Across all feeding groups, mice were provided with a daily allotment of food that exceeded their typical intake, and no group ever consumed the full amount, ensuring that no CR was imposed (Fig. 1a,b and Extended Data Fig. 1)^42^. Any food hoarded within the cage was removed and excluded from Fig. 1b and Extended Data Fig. 2 as described in Methods.

### TRF has sex-specific impacts on longitudinal feeding

Measurements of daily food intake throughout each mouse’s lifespan showed that feeding increased over time in both sexes, then declined near the end of life in male mice, similar to previous mouse studies (Fig. 1a,b and Extended Data Fig. 2)^10,42^. In line with other rodent longitudinal recordings^43^, female mice in our study consumed more food relative to their body weight at older ages than male mice. We also observed sex-specific responses to TRF where 12-h TRF female mice consumed the same amount as AL, whereas 12-h TRF male mice significantly reduced intake compared to AL by 8–14% from 140 to 539 days (~5–18 months) (full details of ANOVA results for all figures can be found in Supplementary Table 1). In contrast, 8-h TRF self-imposed CR for the majority of life in both sexes, with intake in female mice reduced by 10–22% between 140 and 728 days (~5–24 months) and male mice reduced by 9–23% between 140 and 980 days (~5–33 months) relative to sex- and age-matched AL controls. This 8-h TRF schedule, which narrows the daily eating window without explicitly limiting calorie intake as seen in prior mouse and human studies^44,45^, presents an alternative strategy for achieving long-term CR in both sexes.

Although actograms (Fig. 1a) show occasional pellet-taking events outside the defined TRF windows, these largely reflect a technical limitation of the feeding system: pellets dispensed at the very end of the dark phase cannot be retracted, allowing a single pellet to remain available and be retrieved during the light period. Importantly, this represents at most one 300 mg pellet per day, accounting for ≤10% of daily intake in 8-h TRF female (lifetime intake 3.0–5.7 g), ≤8% in 12-h TRF female (3.6–5.9 g), ≤9% in 8-h TRF male (3.2–4.7 g) and ≤8% in 12-h TRF male mice (3.6–5.4 g). We quantitatively assessed this behavior across the full lifespan and found the true average proportion of pellets taken outside the feeding window was low, ranging from 5.17% to 6.34% across feeding groups and sexes (Extended Data Fig. 1b). In addition, food hoarded within the cage accounted for no more than ~10–12% of the amount removed from the feeder (Extended Data Fig. 1c). Together, these data suggest that the majority of food intake in both TRF schedules occurs within the intended feeding window. In contrast, some daytime pellet-taking in AL mice is expected, as C57BL/6 J mice naturally consume a portion of their food during the inactive phase, with ~75% of intake occurring at night under AL conditions^9,10^.

### TRF has sex-specific impacts on longitudinal wheel-running activity

**Extended Data Fig. 3:**
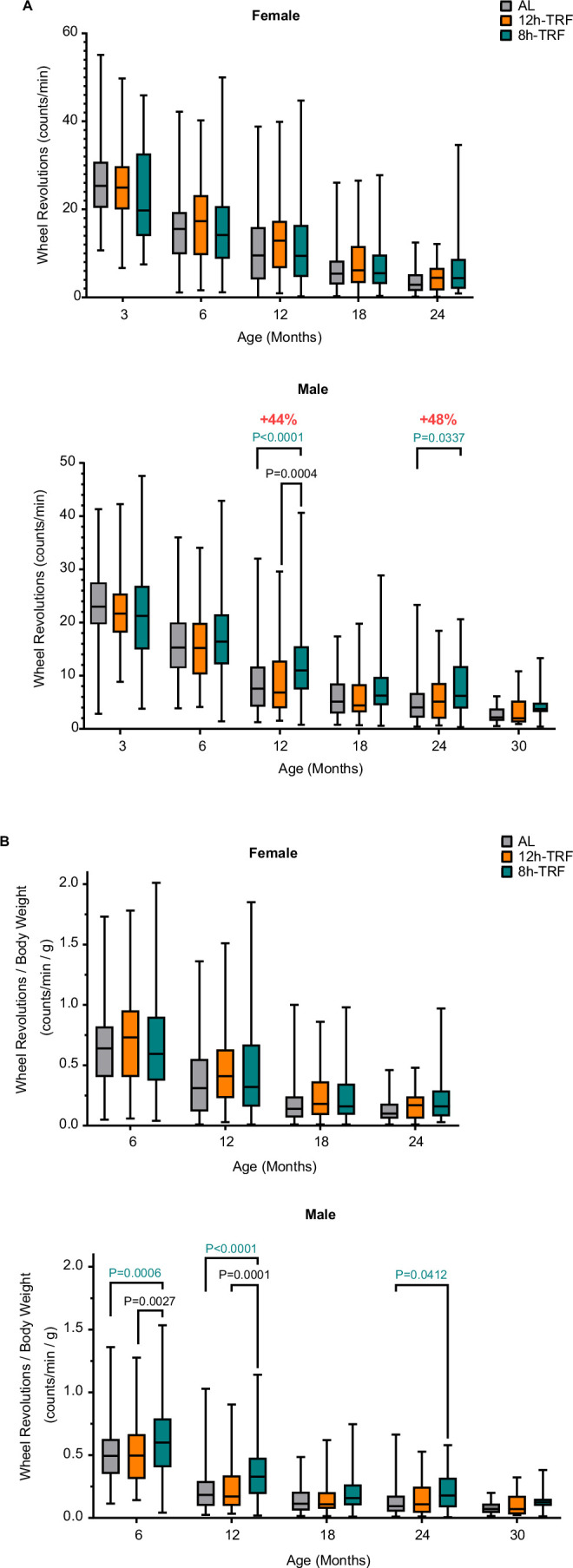
Wheel activity by monthly age epochs. (**a**) In female and male mice, boxplots of daily wheel revolutions (counts/min) at 3 and 6 months of age and then every 6 months following. Wheel running per mouse averaged across 21 days at each age point. Female 3, 6, 12, 18, and 24 months: AL, N=96, 101, 95, 82, 41. 12-h TRF, N=72, 74, 68, 60, 31. 8-h TRF, N=71, 74, 68, 61, 37. Male 3, 6, 12, 18, 24, and 30 months: AL, N=96, 96, 96, 96, 76, 23. 12-h TRF, N=72, 74, 73, 71, 61, 19. 8-h TRF, N=73, 74, 73, 72, 67, 35. (**b**) In female and male mice, boxplots of wheel revolutions (counts/min) relative to body weight (g) at 6 months of age and then every 6 months following. Female 6, 12, 18, and 24 months: AL, N=98, 90, 80, 35. 12-h TRF, N=72, 67, 57, 31. 8-h TRF, N=72, 67, 60, 37. Male 6, 12, 18, 24, and 30 months: AL, N=94, 91, 95, 76, 23. 12-h TRF, N=74, 72, 71, 61, 18. 8-h TRF, N=71, 71, 71, 67, 33. All panels: Box plots indicate the median (center line within box), interquartile range (25th–75th percentiles/bottom-top lines of box), and minimum and maximum values (whiskers). Significant differences (P≤0.05) in TRF vs AL (colored P-values; orange:12-h TRF, teal:8-h TRF) and 12-h vs 8-h TRF (black colored P-values) as determined by two-way ANOVA and Tukey’s post-hoc.

Analysis of daily wheel-running recordings for each mouse revealed that individuals in all feeding groups, of both sexes, consistently displayed nocturnal activity, which subsequently declined with age, as anticipated (Fig. 1a,c and Extended Data Fig. 3)^46^. Only 8-h TRF male mice had increased levels of wheel activity from 224 to 833 days of age (~7–28 months) with the enhanced activity ranging +36–82% from 329 to 833 days (~11–28 months) relative to AL. This male-specific difference further underscores the sex-specific effects of TRF on behavior. Additionally, in line with previous work^10,47^, the sustained increases in activity under 8-h TRF suggest a healthspan-enhancing effect in male mice.

### TRF improves the amplitude of daily rhythmic behaviors

**Fig. 2:**
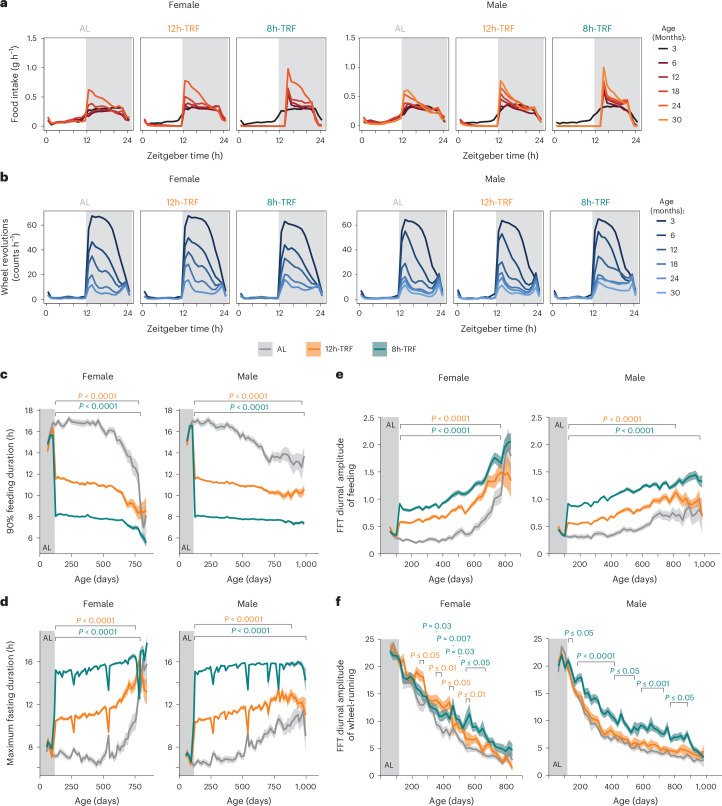
TRF improves the amplitude of daily feeding and wheel-running rhythms. **a**,**b**, 24-h profiles of feeding (**a**) and wheel-running (**b**) starting at 3 months and then every 6 months; 12 h light (white area), 12h dark (gray area). **c**, Longitudinal plots for daily time it takes each group of mice to take 90% of their daily intake from the feeder. **d**, Longitudinal plots for daily maximum fasting times achieved. Large, transient dips in fasting are due to interruptions in data acquisition (for example, fasting blood collection attempt and computer outage). **e**, Longitudinal FFT plots of the diurnal amplitude of feeding with age. **f**, Longitudinal FFT plots of the diurnal amplitude of wheel-running with age. In **c**–**f**, AL baseline feeding (gray area); TRF feeding conditions (white area). Each point represents mean of 14 days ± s.e.m. (shaded regions). Age blocks with significant differences (*P* ≤ 0.05) in TRF versus AL (colored *P* values; orange: 12-h TRF, teal: 8-h TRF) marked by brackets/dots as determined by type III ANOVA (Wald χ^2^) and Holm’s post hoc. Starting numbers of mice for feeding/wheel profiles: female: AL, *n* = 103/96; 12-h TRF, *n* = 76/73; 8-h TRF, *n* = 76/71; male: AL, *n* = 97/96; 12-h TRF, *n* = 72/72; 8-h TRF, *n* = 71/73.

We next examined age-related changes in the diurnal profiles of feeding and wheel-running, two well-established rhythmic behaviors, as high-amplitude behavioral rhythms are closely associated with improved health outcomes (Fig. 2a,b)^18,19,48,49^. As intended, TRF reduced the feeding window, shown by longitudinal measurements of the time required for each group to retrieve 90% of their daily food intake from the feeder. Across both sexes, mice under 12-h TRF took 90% of their food within 12 h, whereas those under 8-h TRF did so within 8 h (Fig. 2c). The time required to reach this threshold declined with age across groups, particularly in female mice, indicating that feeding became more consolidated in older animals (Fig. 2a,c). Consistently, both TRF groups showed significantly longer daily maximum fasting durations than AL controls, exceeding 10 h in the 12-h TRF group and 14 h in the 8-h TRF group across both sexes (Fig. 2d).

A fast Fourier transform (FFT) analysis of diurnal amplitude, reflecting the strength of daily rhythms, confirmed that TRF increased feeding amplitude and led to a preservation of enhanced rhythmicity with age, particularly in the 8-h TRF group (Fig. 2a,e). Although the diurnal amplitude of wheel-running declined with age across all groups, 8-h TRF male mice maintained stronger rhythms throughout life compared to controls (Fig. 2b,f). In female mice, TRF induced intermittent enhancements in wheel amplitude relative to controls. As mice age, TRF, particularly an 8 h window, counteracted the natural decline in behavioral rhythmicity by consolidating food intake and preserving higher-amplitude wheel running activity compared with AL controls.

### TRF improves body weight and composition

**Fig. 3:**
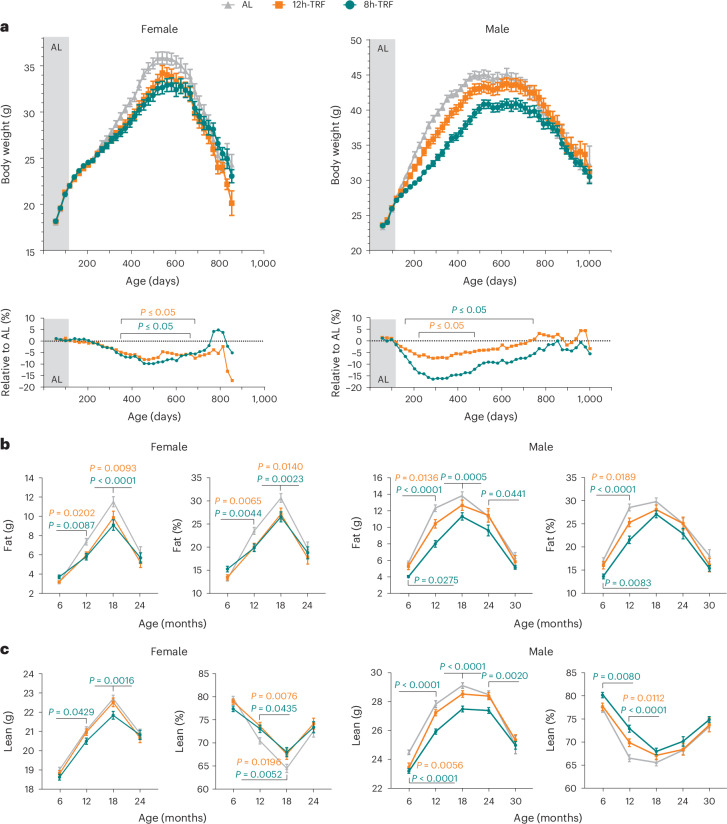
A 12-h TRF window is sufficient to reduce body weight and fat mass in both sexes. **a**, Longitudinal profiles of body weight (top) and percent differences in body weight (bottom) relative to AL controls with age. AL baseline feeding (gray area); TRF feeding conditions (white area). Each point represents mean of 21 days (cage change) ± s.e.m. (bars). Age blocks with significant differences (*P* ≤ 0.05) in TRF versus AL marked by brackets as determined by two-way ANOVA and Tukey’s post-hoc. Starting number of mice: female: AL, *n* = 103; 12-h TRF, *n* = 76; 8-h TRF, *n* = 76; male: AL, *n* = 102; 12-h TRF, *n* = 77; 8-h TRF, *n* = 76. **b**, Total fat mass in g and as a percentage of body weight with age. **c**, Total lean mass in g and as a percentage of body weight with age. **b**,**c**, Means every 6 months ± s.e.m. (bars). Significant differences (*P* ≤ 0.05) in TRF versus AL (colored *P* values; orange: 12-h TRF, teal: 8-h TRF) as determined by two-way ANOVA and Tukey’s post-hoc. Female 6, 12, 18 and 24 months: AL, *n* = 101, 91, 82, 43; 12-h TRF, *n* = 70, 68, 58, 32; 8-h TRF, *n* = 72, 68, 61, 38. Male 6, 12, 18, 24 and 30 months: AL, *n* = 102, 96, 97, 77, 23; 12-h TRF, *n* = 76, 77, 72, 62, 19; 8-h TRF, *n* = 75, 73, 72, 67, 36.

**Extended Data Fig. 4:**
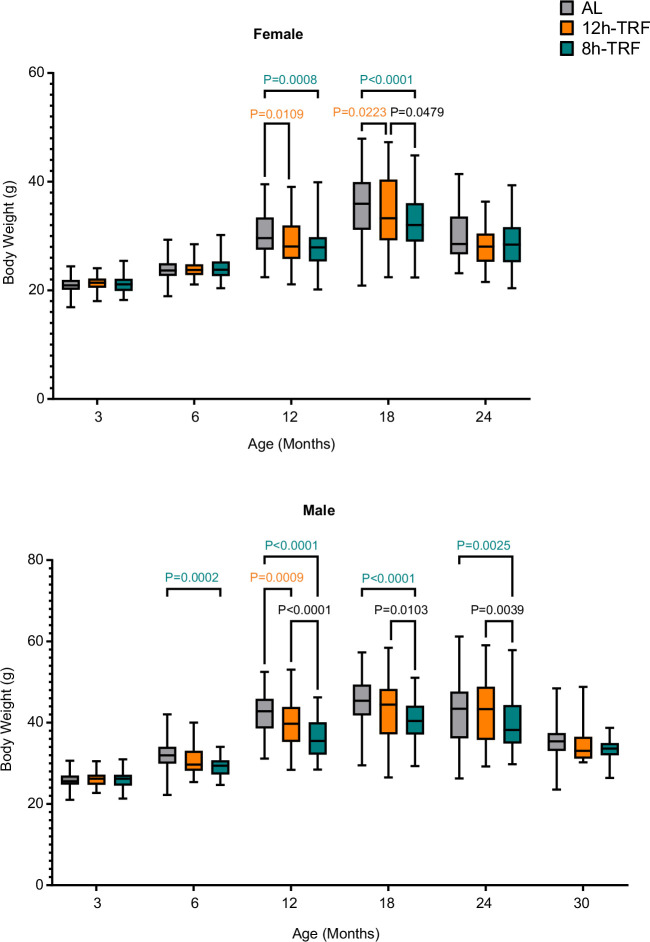
Body weight by monthly age epochs. In female and male mice, boxplots of body weight (g) at 3 and 6 months of age and then every 6 months following. Box plots indicate the median (center line within box), interquartile range (25th–75th percentiles/bottom-top lines of box), and minimum and maximum values (whiskers). Significant differences (P≤0.05) in TRF vs AL (colored P-values; orange:12-h TRF, teal:8-h TRF) and 12-h vs 8-h TRF (black colored P-values) as determined by two-way ANOVA and Tukey’s post-hoc. Female 3, 6, 12, 18, and 24 months: AL, N=103, 101, 95, 82, 39. 12-h TRF, N=76, 74, 69, 57, 31. 8-h TRF, N=76, 74, 68, 61, 37. Male 3, 6, 12, 18, 24, and 30 months: AL, N=102, 102, 100, 96, 75, 21. 12-h TRF, N=77, 77, 76, 72, 61, 16. 8-h TRF, N=76, 74, 75, 72, 67, 32.

Body weights were recorded every 21 days (Fig. 3a and Extended Data Fig. 4), and body composition was analyzed every 6 months using nuclear magnetic resonance imaging (EchoMRI, Houston, TX) (Fig. 3b,c). In female mice, 12-h TRF without CR lessened age-related gains in body weight by 5–8% from 350–686 days of age (~12–23 months) compared with AL controls. This feeding restriction also improved fat vs lean body composition, reducing fat mass while increasing lean mass by 3–4% at 12 and 18 months of age. Unexpectedly, 8-h TRF female mice with self-imposed CR did not exhibit any strong improvements beyond 12-h TRF in weight or fat versus lean composition. In male mice, 12-h TRF reduced body weight gain by 5–7% from 224 to 476 days (~7–16 months) and improved fat versus lean composition by 3% at 12 months. 8-h TRF in male mice enhanced these benefits, lowering body weight gain by 5–16% from 161 to 749 days (~5–25 months) and improving fat versus lean composition by 3–7% from 6 to 12 months. Ultimately, these results demonstrate that although 12-h TRF is sufficient to attenuate weight gain and optimize body composition in both sexes, the benefits are more prolonged in female mice; conversely, male mice derive significantly greater advantages from a more restrictive 8-h TRF schedule combined with self-imposed CR.

### TRF reshapes diurnal RER and EE rhythms in a time-dependent manner

Metabolic changes under TRF were assessed using indirect calorimetry at 19 months of age. Mice were randomly selected and individually housed in Promethion metabolic cages equipped with automated feeders for 5–6 days at room temperature (25 °C), followed by 5 days at thermoneutrality (30 °C), to assess metabolic parameters independent of the energetic demands of thermoregulation (Supplementary Figs. 1 and 2). As expected, 24-h respiratory exchange ratio (RER) profiles exhibited diurnal variation with a significant TRF x time of day interaction (Supplementary Fig. 1a). Although TRF did not have a significant main effect on mean 24-h RER, post hoc analyses showed 8-h TRF mice had lower RER during the delayed feeding onset in both sexes, consistent with prolonged lipid oxidation, and higher nocturnal RER at select time points in male mice at room temperature, suggesting increased carbohydrate utilization and greater diurnal amplitude.

The 24-h energy expenditure (EE) profiles also varied diurnally and were impacted by TRF in female mice, with 8-h TRF trending lower during the rest phase (Supplementary Fig. 1b). At thermoneutrality, daytime EE was significantly reduced at several time points in 8-h TRF female mice, whereas effects in male mice were minimal and limited to light/dark and feeding/fasting transitions. Impacts on EE were further analyzed by ANCOVA to account for body composition (Supplementary Fig. 2). Homogeneity of regression slopes was confirmed, and although lean mass significantly influenced outcomes in male mice, TRF effects on EE remained largely time-specific rather than altering overall 24-h EE in either sex.

### Glucose homeostasis is moderately improved by 8-h TRF in male mice

We assessed glucose homeostasis by measuring 12-h fasting glucose and performing glucose tolerance tests at ZT10 at 6, 12 and 18 months of age. Neither 12-h TRF nor 8-h TRF produced consistent reductions in fasting glucose across all ages, as seen in our previous study (Supplementary Fig. 3a)^9^. In female mice, 8-h TRF lowered fasting glucose at 12 months but increased it at 18 months. Given that fasting glucose typically declines with age in mice, this increase may reflect a healthier metabolic state^50^. Glucose tolerance tests also revealed sex-specific effects. 8-h TRF female mice showed modest improvement at 12 months only, whereas male mice exhibited improvements at 6 months in both TRF regimens, with smaller benefits persisting at 12 months in 8-h TRF (Supplementary Fig. 3b,c). No effects were observed at 18 months, and fasting insulin levels were unchanged across all groups (Supplementary Fig. 4).

### Little to no impact on circulating metabolic and inflammatory markers

To determine whether TRF alters systemic metabolic or inflammatory signaling, we measured circulating levels of leptin, brain-derived neurotrophic factor (BDNF), monocyte c chemoattractant protein-1 (MCP-1), tumor necrosis factor α (TNFα), interleukin-1β (IL-1β), IL-6 and IL-10 from 12-h fasted plasma collected at ZT10 at 6, 12 and 18 months of age. Overall, TRF had minimal effects, with no sustained differences across ages (Supplementary Fig. 4). At 6 months, 8-h TRF male mice showed elevated BDNF and MCP-1, but these differences were not maintained with age. At 18 months, 12-h TRF male mice exhibited higher IL-1β, whereas 8-h TRF female mice showed increased MCP-1, suggesting some age- and sex-specific effects of TRF on inflammatory markers.

### TRF slows age-related increases in frailty index values

**Fig. 4:**
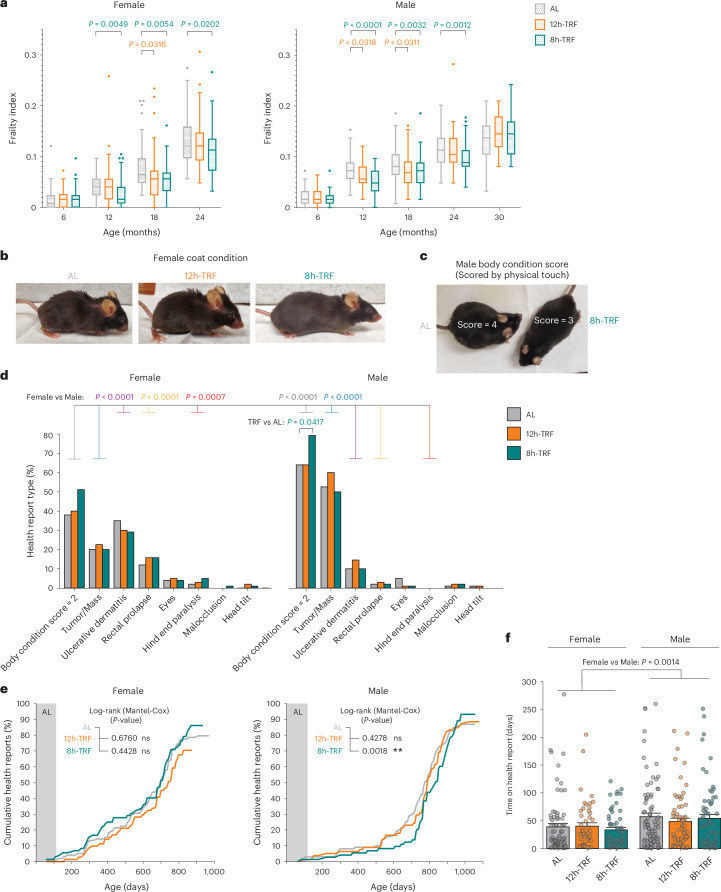
TRF reduces frailty index in both sexes but only delays onset of health reports in male mice. **a**, Boxplots of frailty index scores every 6 months. Boxplots indicate the median (center line in box), interquartile range (IQR; 25th–75th percentiles/bottom-top lines of box), and whiskers extending to the most extreme values within 1.5x IQR; outliers are plotted individually. Significant differences (*P* ≤ 0.05) in TRF versus AL (colored *P* values; orange: 12-h TRF, teal: 8-h TRF) as determined by two-way ANOVA and Tukey’s post-hoc. Female 6, 12, 18 and 24 months: AL, *n* = 79, 71, 63, 44; 12-h TRF, *n* = 74, 69, 60, 34; 8-h TRF, *n* = 74, 67, 61, 40. Male 6, 12, 18, 24 and 30 months: AL, *n* = 79, 77, 73, 55, 23; 12-h TRF, *n* = 78, 77, 74, 63, 22; 8-h TRF, *n* = 76, 75, 72, 67, 39. **b**, One example parameter contributing to reduced frailty index in 8-h TRF female mice: coat condition, *P* < 0.001 at 24 months compared to AL (full list, see Extended Data Figs. 5 and 6). **c**, One example parameter contributing to reduced frailty index in 8-h TRF male mice: BCS, *P* < 0.001 12*–*18 months compared to AL. (Full list see Extended Data Figs. 7 and 8). **d**, Percentage of mice in each feeding group and sex reported to veterinary staff for BCS = 2, tumor/mass, ulcerative dermatitis, rectal prolapses, eye ulcerations/swelling, hind end paralysis, malocclusions and head tilts. Significant differences (*P* ≤ 0.05) in TRF versus AL or female versus male determined by contingency tables and two-sided Fisher’s exact tests. **e**, Cumulative curve showing the percentage of veterinary health reports filed in each group with age. Two-sided log-rank Mantel-Cox test. **f**, Time spent on health report until death. Two-way ANOVA and Holm’s post hoc. Data shown as mean ± s.e.m. In **d**–**f**: female: AL, *n* = 98; 12-h TRF, *n* = 71; 8-h TRF, *n* = 72. Male: AL, *n* = 99; 12-h TRF, *n* = 78; 8-h TRF, *n* = 73.

**Extended Data Fig. 5:**
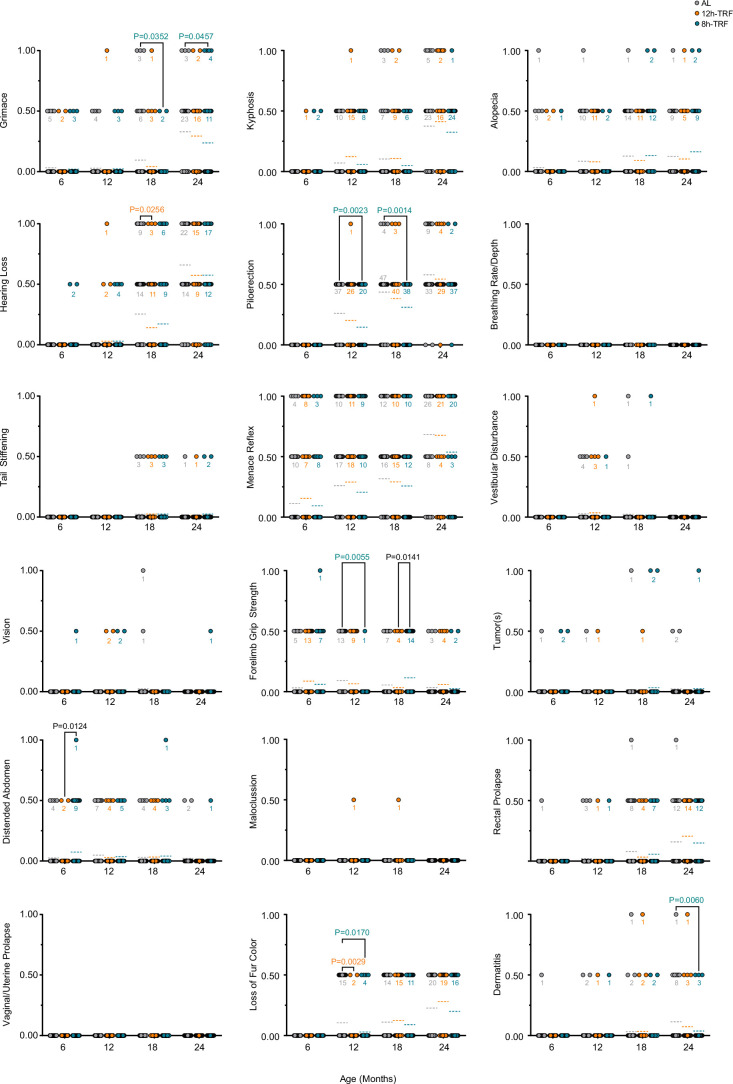
Individual frailty parameter scores in females Part 1. Dot plots of frailty scores every 6 months of age in female mice for 18 of the 31 parameters modified from^51^. Parameters scored as 0 for no, 0.5 for mild, and 1 for severe deficit. N values shown in graph for any mice with scores >0. Mean shown as dashed line. Significant differences (P≤0.05) in TRF vs AL (colored P-values; orange:12-h TRF, teal:8-h TRF) and 12-h vs 8-h TRF (black colored P-values) as determined by two-way ANOVA and Tukey’s post-hoc. Female 6, 12, 18, and 24 months: AL, N=79, 71, 63, 44. 12-h TRF, N=74, 69, 60, 34. 8-h TRF, N=74, 67, 61, 40.

**Extended Data Fig. 6:**
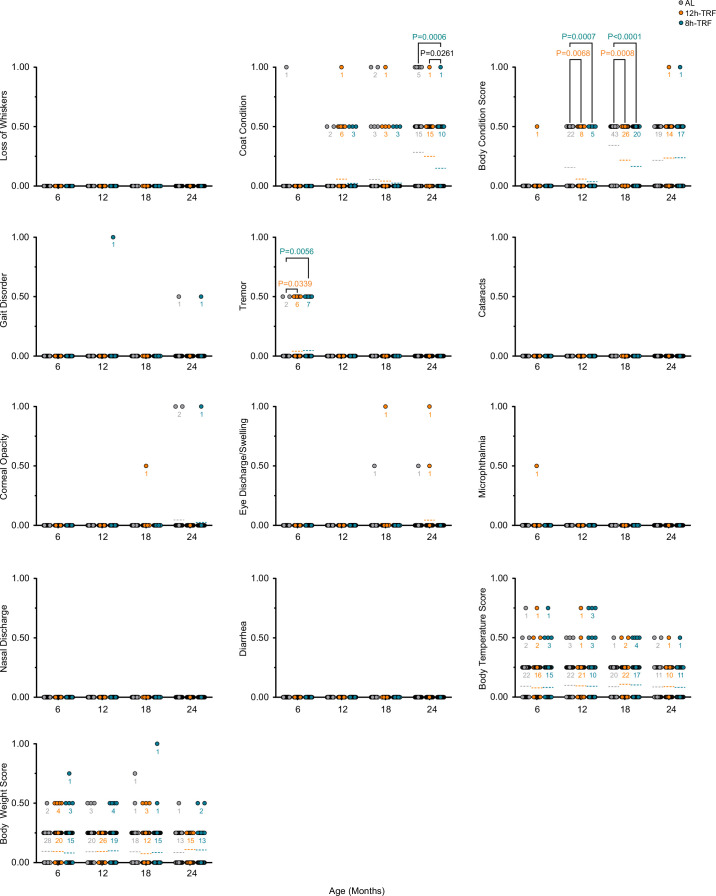
Individual frailty parameter scores in females Part 2. Dot plots of frailty scores every 6 months of age in female mice for 13 of the 31 parameters modified from^51^. Most parameters scored as 0 for no, 0.5 for mild, and 1 for severe deficit. Body temperature and body weight scores were scored 0, .25, .5, .75, or 1 with higher values representing more severe deviation of the parameter from the group mean. N values shown in graph for any mice with scores >0. Mean shown as dashed line. Significant differences (P≤0.05) in TRF vs AL (colored P-values; orange:12-h TRF, teal:8-h TRF) and 12-h vs 8-h TRF (black colored P-values) as determined by two-way ANOVA and Tukey’s post-hoc. Female 6, 12, 18, and 24 months: AL, N=79, 71, 63, 44. 12-h TRF, N=74, 69, 60, 34. 8-h TRF, N=74, 67, 61, 40.

**Extended Data Fig. 7:**
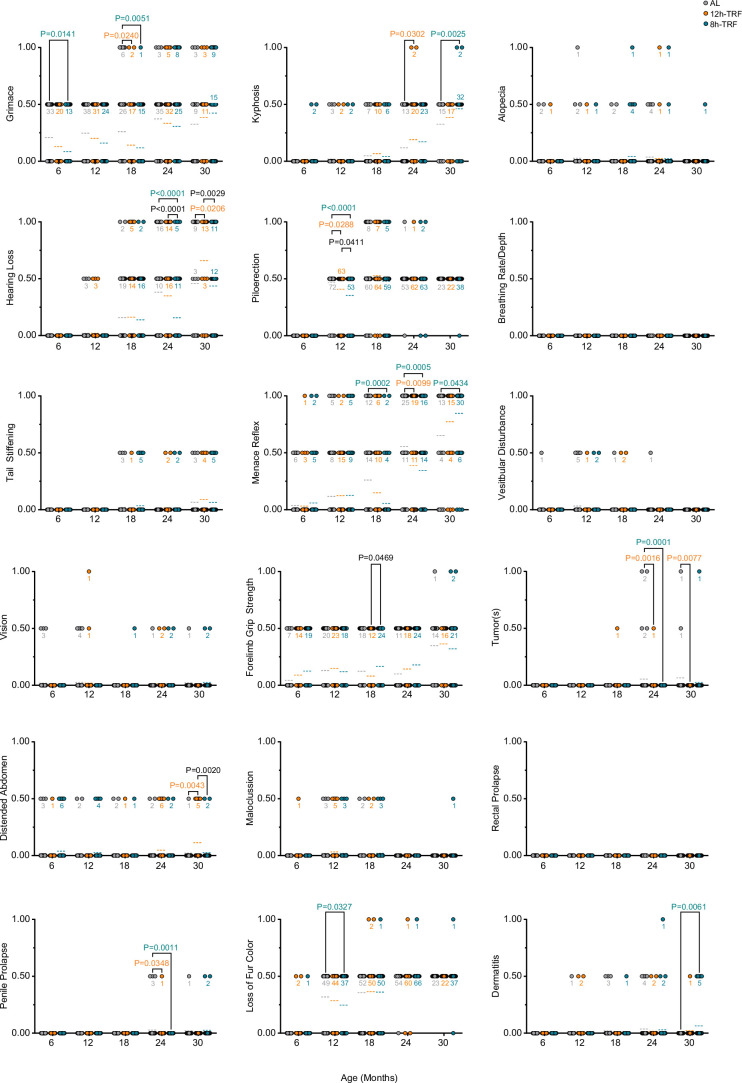
Individual frailty parameter scores in males Part 1. Dot plots of frailty scores every 6 months of age in male mice for 18 of the 31 parameters modified from^51^. Parameters scored as 0 for no, 0.5 for mild, and 1 for severe deficit. N values shown in graph for any mice with scores >0. Mean shown as dashed line. Significant differences (P≤0.05) in TRF vs AL (colored P-values; orange:12-h TRF, teal:8-h TRF) and 12-h vs 8-h TRF (black colored P-values) as determined by two-way ANOVA and Tukey’s post-hoc. Male 6, 12, 18, 24, and 30 months: AL, N=79, 77, 73, 55, 23. 12-h TRF, N=78, 77, 74, 63, 22. 8-h TRF, N=76, 75, 72, 67, 39.

**Extended Data Fig. 8:**
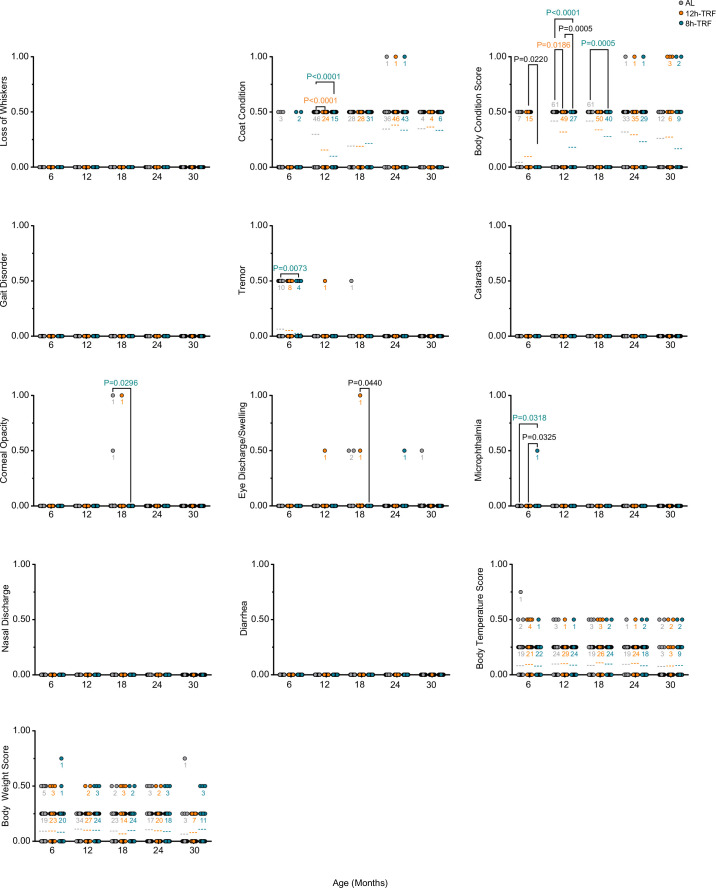
Individual frailty parameter scores in males Part 2. Dot plots of frailty scores every 6 months of age in male mice for 13 of the 31 parameters modified from^51^. Most parameters scored as 0 for no, 0.5 for mild, and 1 for severe deficit. Body temperature and body weight scores were scored 0, .25, .5, .75, or 1 with higher values representing more severe deviation of the parameter from the group mean. N values shown in graph for any mice with scores >0. Mean shown as dashed line. Significant differences (P≤0.05) in TRF vs AL (colored P-values; orange:12-h TRF, teal:8-h TRF) and 12-h vs 8-h TRF (black colored P-values) as determined by two-way ANOVA and Tukey’s post-hoc. Male 6, 12, 18, 24, and 30 months: AL, N=79, 77, 73, 55, 23. 12-h TRF, N=78, 77, 74, 63, 22. 8-h TRF, N=76, 75, 72, 67, 39.

Frailty, a widely used measure of healthspan in aging mice, integrates 31 physiological and behavioral parameters into a composite index^51^. Frailty assessments were conducted every 6 months and revealed that both 12-h and 8-h TRF significantly reduced frailty index scores at specific ages compared to AL controls (Fig. 4a–c and Extended Data Figs. 5–8). In female mice, 12-h TRF significantly reduced frailty index scores at 18 months versus AL, with improvements in hearing loss, loss of fur color, tremoring and body condition score (BCS) individual assessments defining the key differences across multiple ages. 8-h TRF further extended reductions in frailty index from 12 to 24 months, with specific improvements observed in grimace, piloerection, forelimb grip strength, coat condition, tremoring, ulcerative dermatitis and BCS. Male mice showed similar trends: 12-h TRF reduced frailty indices between 12 and 18 months, primarily improving grimace, menace reflex, piloerection, tumors, penile prolapses, coat condition and BCS, whereas 8-h TRF sustained these benefits through 24 months and improved grimace, menace reflex, hearing loss, piloerection, tumors, penile prolapses, loss of fur color, coat condition, BCS, tremoring and corneal opacity. These results show that TRF significantly lowers age-related frailty in a dose- and sex-dependent manner, with 8-h TRF providing prolonged benefits and improving the greatest number of health measures.

### The age-related onset of health reports reveals sex-specific differences and a delayed onset in 8-h TRF male mice

As mice developed health issues they were reported to veterinary staff for more frequent monitoring. These reports revealed that TRF did not reduce the relative frequency of commonly reported ailments compared to AL (Fig. 4d). Indeed, 8-h TRF male mice had significantly more reports of BCSs declining to 2 (scored 1–5 (1 emaciated, 2 underconditioned, 3 ideal condition, 4 overconditioned, 5 obese)). However, we observed a clear sex-dependent pattern in the types and frequencies of reported conditions. Female mice, regardless of feeding group, were significantly more likely to be reported for ulcerative dermatitis, rectal prolapse and hind limb paralysis. In contrast, male mice were more frequently reported for external tumors and abdominal masses, as well as low BCS (2). The onset of age-related health issues also differed by sex, with 8-h TRF delaying the median health report onset by 52 days in male mice only compared to AL controls (Fig. 4e). Additionally, although there were no significant differences under TRF, overall, female mice overall spent significantly fewer days on health report before death than male mice (Fig. 4f). Altogether this demonstrates there is sex specificity in the development of common husbandry-related diseases.

### TRF has minimal impacts on hematological markers

To determine whether lifelong TRF impacts hematological markers of systemic health and aging^52^, we measured complete blood counts at 6, 12 and 18 months of age. Red blood cell (RBC) counts, hemoglobin, hematocrit, red cell indices (mean corpuscular volume (MCV), mean corpuscular hemoglobin (MCH) and mean corpuscular hemoglobin concentration), platelet counts and white blood cells counts were largely unchanged across feeding regimens (Supplementary Figs. 5–7). An exception was observed in 18-month-old female mice, where 8-h TRF increased RBC counts with reduced MCV and MCH (that is, smaller and likely older RBCs) compared to AL controls (Supplementary Fig. 5a). As aging in mice is commonly associated with reduced RBC survival^53^, this pattern may reflect improved RBC maintenance rather than a pathological shift. Importantly, mean corpuscular hemoglobin concentration, hemoglobin and hematocrit levels were unchanged in this group, indicating preserved oxygen-carrying capacity.

### 8-h TRF extends lifespan in male mice only

**Fig. 5:**
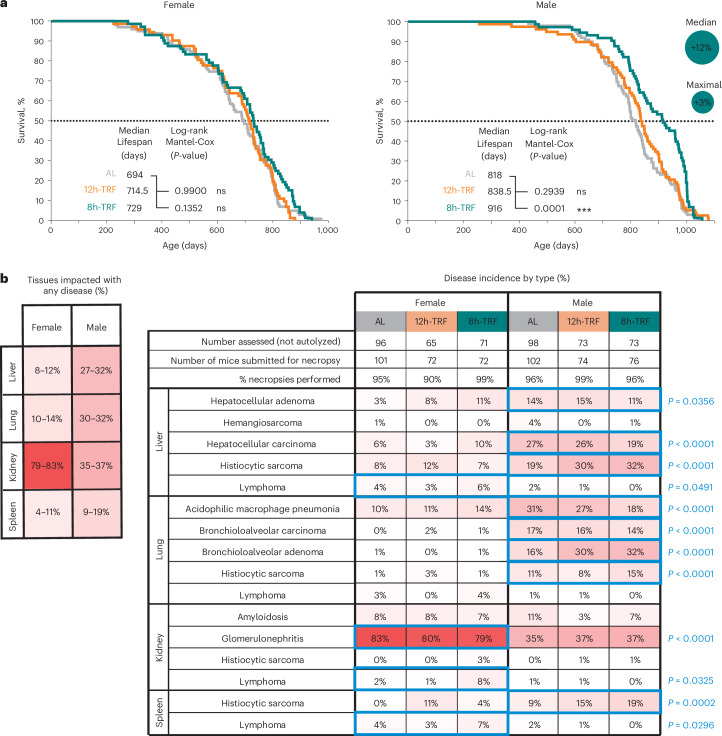
8-h TRF extends lifespan in male mice. **a**, Kaplan-Meier survival curves and day median lifespan reached. Two-sided log-rank Mantel-Cox test for significant difference (*P* ≤ 0.05) in overall survival TRF versus AL. Two-sided Fisher’s exact test for maximal survival TRF versus AL. Female: AL, *n* = 99; 12-h TRF, *n* = 72; 8-h TRF, *n* = 72. Male: AL, *n* = 99; 12-h TRF, *n* = 78; 8-h TRF, *n* = 73. **b**, Histopathology results showing the percentage incidence of any disease type in the most impacted tissue types between female and male mice (left), and incidence by specific disease type within these tissues separated by sex and feeding group (right). Full table with all tissues and diseases is shown in Supplementary Table 3. Significant differences (*P* ≤ 0.05) in female versus male highlighted by blue boxes as determined by contingency tables and two-sided Fisher’s exact tests.

**Extended Data Fig. 9:**
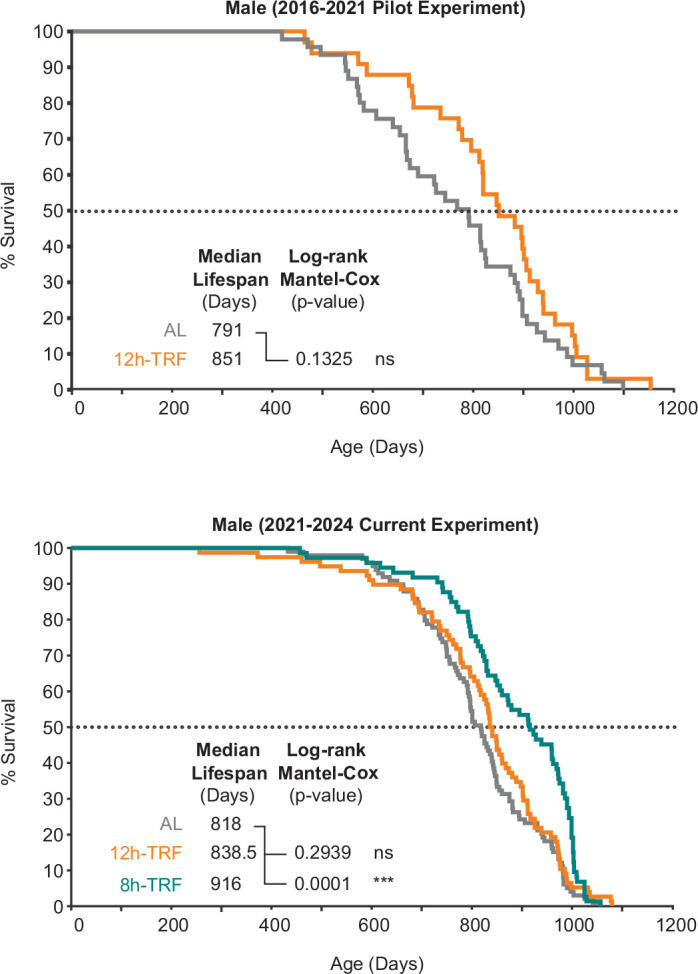
Repeat experiments show 12-h TRF does not extend lifespan in males. Kaplan- Meier survival curves and day median lifespan reached in a pilot experiment vs our current experiment. Log-Rank Mantel-Cox Test for significant difference (P≤0.05) in overall survival TRF vs AL. Pilot experiment: AL, N=43. 12-h TRF, N=33. Current experiment: AL, N=99. 12-h TRF, N=78. 8-h TRF, N=73.

Here, we were able to investigate the impact of circadian aligned TRF with regular chow diet, on lifespan in mice (Fig. 5a and Supplementary Table 2). In female mice, no significant extension of median lifespan was observed with either 12-h TRF or 8-h TRF. Median survival was 715 days and 729 days, respectively, compared to 694 days in AL controls. In male mice, 12-h TRF also failed to extend lifespan, with a median survival of 839 days compared to 818 days in AL, a finding now reproduced across two independent experiments (Extended Data Fig. 9). Interestingly, 8-h TRF with lifelong self-imposed CR significantly improved overall survival for male mice. Median lifespan increased by approximately 12%, reaching 916 days, and maximal lifespan was prolonged by ~3%. Although TRF is beneficial for improving health in both sexes, its capacity to extend lifespan appears to be feeding window and sex dependent.

### Sex-dependent differences in disease types and incidence at death

After death, each mouse underwent gross necropsy and histopathological analysis by a veterinary pathologist to determine the types of diseases that contributed to its debilitation (Fig. 5b and Supplementary Table 3). As shown previously^10^, the types and relative frequency of diseases were similar for male mice across all feeding conditions, with the most common cause of death or debilitation being neoplasia (Fig. 5b and Supplementary Table 4 for all Fisher’s exact tests). Along with the delayed onset of health reports, overall, this finding supports that rather than preventing disease, 8-h TRF delays the development of age-related diseases in male mice.

TRF did not reduce the overall occurrence of disease in female mice either. However, female mice developed neoplasms far less frequently than male mice, such as benign adenomas, malignant carcinomas and histiocytic sarcoma in the liver and lung, acidophilic macrophage pneumonia in the lung and histiocytic sarcoma in the spleen. Instead, most female mice were debilitated by glomerulonephritis in the kidneys and had a higher incidence of lymphoma than male mice. These results have revealed pronounced differences in neoplasia and development of other age-related morbidities between the sexes.

However, TRF altered the incidence of a few specific neoplastic subtypes. In male mice, bronchioloalveolar adenomas increased in both TRF groups relative to AL controls, whereas in female mice, hepatocellular adenomas were more frequent under 8-h TRF. Despite this increase in lesions with malignant potential, the absence of more carcinomas suggests that TRF may shift pathology toward less aggressive neoplastic lesions rather than fully prevent tumorigenesis.

### TRF extends healthspan, with more prolonged benefits in female mice

**Fig. 6:**
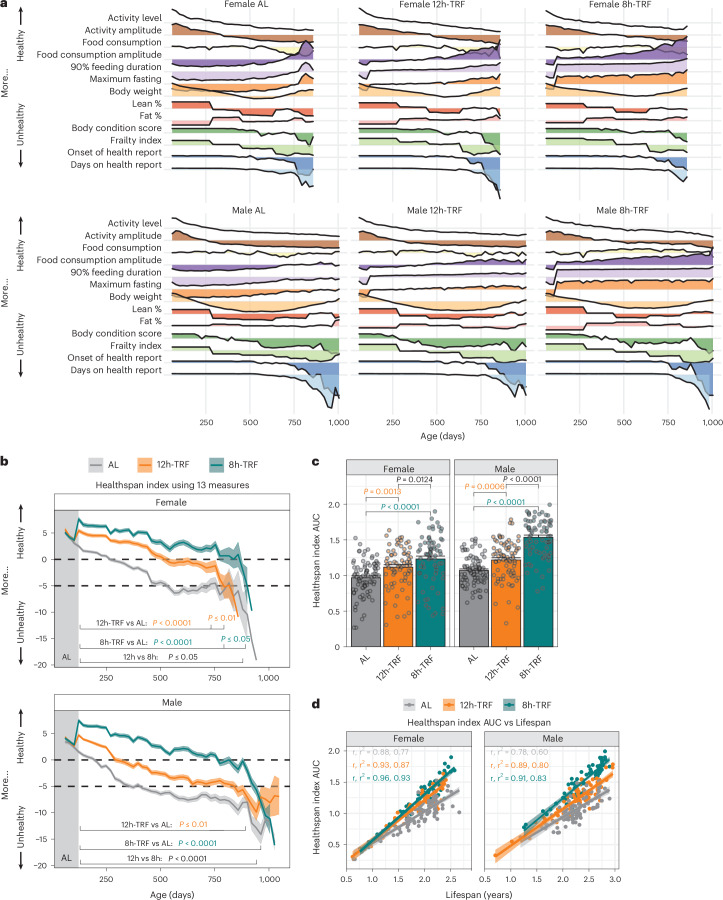
TRF improves healthspan in both sexes. **a**, Ridgeline plots for each of the 13 health measures recorded throughout the experiment. All parameters were standardized (mean = 0, SD = 1) and, where appropriate, oriented in the positive direction to reflect better health. **b**, Healthspan index summing the 13 health measures for each group from panel **a**. Higher values indicate better health and lower values poorer health. Dashed lines (black) mark the age at which each group’s index crosses subjective thresholds of 0 and –5. Each point represents mean of 21 days ± s.e.m. (shaded regions). Age blocks with significant differences (*P* ≤ 0.05) in TRF versus AL (colored *P* values; orange: 12-h TRF, teal: 8-h TRF) and 12-h versus 8-h TRF (black *P* values) marked by brackets as determined by type III ANOVA (Wald χ^2^) and Holm’s post hoc. **c**, Area under the curve (AUC) for healthspan index in female and male mice. Mean ± s.e.m. (bars). Significant differences as determined by type III ANOVA and Holm’s post hoc. **d**, Correlation plots of healthspan AUC versus lifespan in years. Regression lines are displayed and shaded areas represent s.e.m. Correlation coefficients (r) and coefficients of determination (r^2^) are shown. For all panels, female: AL, *n* = 103; 12-h TRF, *n* = 77; 8-h TRF, *n* = 77. Male: AL, *n* = 104; 12-h TRF, *n* = 78; 8-h TRF, *n* = 77.

Although TRF improved and attenuated age-related changes in several markers of health, we next aimed to quantify both the magnitude and duration of its overall impact by measuring healthspan. Frailty index is commonly used to determine healthspan^51,54^, but it cannot incorporate the impact of behavioral changes in wheel-activity and food consumption and can miss important physiological changes in the time between assessments. To generate a continuous healthspan index, we integrated frailty with 12 additional health measures by first normalizing each data set (mean = 0, standard deviation = 1), then scaling the parameter directionally by providing context based on whether higher or lower values reflected better health (for example, high wheel-running activity was scaled positively, whereas low frailty scores were inverted and also scaled positively) (Fig. 6a), and finally summing the values across age points (Fig. 6b).

With higher values reflecting better health, we found that both female and male mice under TRF had significantly improved healthspan indices throughout life compared to AL controls (Fig. 6b, c). In female mice, benefits persisted until 774 days (~26 months) under 12-h TRF and 879 days (~29 months) under 8-h TRF, with 8-h TRF outperforming 12-h TRF until 858 days (~29 months). In male mice, benefits persisted until 879 days (~29 months) under 12-h TRF and 963 days (~32 months) under 8-h TRF, with 8-h TRF outperforming 12-h TRF until 942 days (~31 months). Importantly, healthspan indices strongly correlated with lifespan in both sexes, demonstrating that this integrated metric captures biologically meaningful health outcomes (Fig. 6d).

These health declines were delayed more in female than in male mice, particularly under TRF. Using subjective thresholds of 0 or −5 (black dashed lines), AL female mice crossed the −5 threshold more than 100 days later than AL male mice, and under 12-h TRF, female mice crossed the 0 threshold more than 200 days later than male mice on the same regimen. Although 8-h TRF extended time above the 0 threshold by ~500 days relative to AL in both sexes, this represented a larger proportion of lifespan in female mice. Together, these findings show that TRF extends healthspan in both sexes and suggest that, even without lifespan extension, female mice remain healthier for a greater proportion of life than male mice.

**Extended Data Fig. 10:**
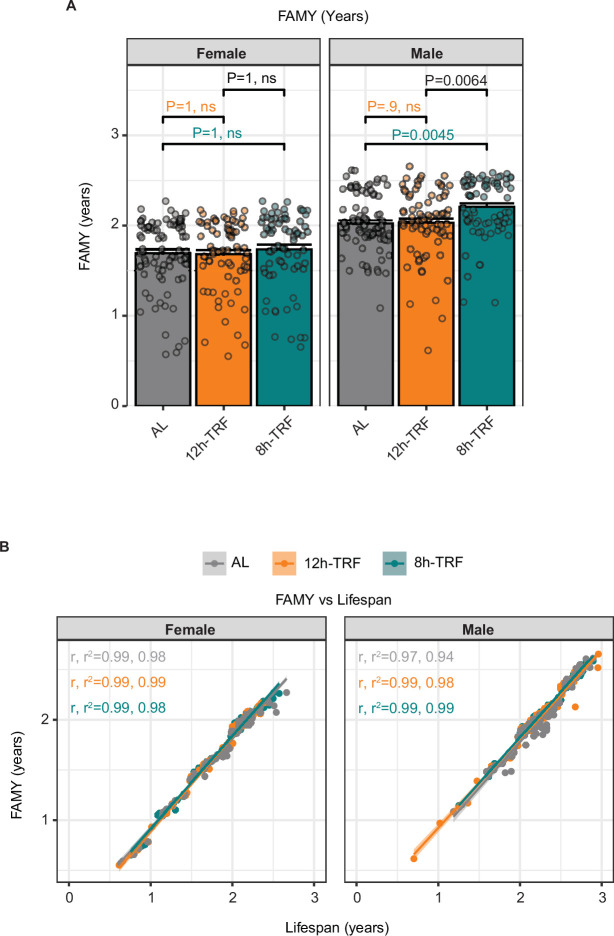
Healthspan as measured by Frailty-Adjusted Mouse Years (FAMY). In female and male mice: (**a**) FAMY (years) is another method to measure healthspan which utilizes frailty scores as described in^55^. Higher FAMY = more healthy years. Mean ± SEM (bars). Significant differences (P≤0.05) in TRF vs AL (colored P-values; orange:12-h TRF, teal:8-h TRF) and 12-h vs 8-h TRF (black colored P-values) as determined by Type III ANOVA and Holm’s post-hoc. (**b**) Correlation plots of FAMY vs lifespan in years. Regression lines are displayed and shaded areas represent SEM. Correlation coefficients (r) and coefficients of determination shown (r^2^). For all panels: Female: AL, N=103. 12-h TRF, N=77. 8-h TRF, N=77. Male: AL, N=104. 12-h TRF, N=78. 8-h TRF, N=77.

To validate our findings of TRF-induced healthspan extension further, we also applied a previously published summary metric, frailty-adjusted mouse years (FAMY)^55^, which relies solely on frailty scores. FAMY similarly detected a significant healthspan increase in 8-h TRF male mice (Extended Data Fig. 10a). The lower frequency of frailty assessments in our study (once every 6 months) likely contributed to the banded distribution of FAMY scores and may explain differences between methods. Nevertheless, FAMY strongly correlated with lifespan across all groups (Extended Data Fig. 10b) supporting the robustness of our findings and suggesting that our higher-resolution composite index may be more sensitive for detecting healthspan changes in female mice.

## Discussion

Previous studies have established that TRF improves circadian rhythms, ameliorates metabolic syndrome, and improves overall health in obesity models consuming a ‘Western diet’ high in fats and sugars^27–30^. We expand on these findings by revealing that circadian aligned TRF confers significant, long-term health benefits even under normal nutritional conditions. We show that, depending on the feeding window and sex, TRF prolonged higher amplitudes of daily rhythms in feeding and wheel-running, which are known to feed back on the circadian clock to drive robust molecular and behavioral oscillations^13,14,16,45,56,57^. Both TRF paradigms were sufficient to significantly reduce age-related increases in weight and fat mass, having the largest impact during midlife. TRF similarly slowed the age-related decline in lean composition, which may also contribute to the observed improvements in adiposity and suggests that TRF can delay the onset of functional disabilities^58,59^. We also present findings that both TRF windows reduce frailty index in female and male mice, as in CR and intermittent fasting studies^60–63^. Veterinary health reports were largely sex-dependent, and overall TRF did not alter the types of deficits observed. Notably, 8-h TRF male mice displayed a higher proportion of low BCSs (score of 2), but this occurred alongside a delayed onset of health issues and overall increased lifespan and likely reflects the survival of more mice to advanced ages rather than a detrimental effect of TRF. Consistently, no differences in BCS were observed at 30 months in male mice within the individual frailty assessments. Altogether, this demonstrates that TRF does not merely act as a remedial strategy to ‘rescue’ poor health but can actively improve behavioral and physiological markers of health for both sexes under normal metabolic aging conditions.

Despite these robust improvements in health, TRF-induced reductions in body weight and adiposity were not accompanied by consistent improvements in glucose tolerance or sustained alterations in fasting glucose, insulin, leptin, or circulating inflammatory markers. These findings indicate that the metabolic benefits of TRF do not require large shifts in systemic endocrine or inflammatory signaling. Instead, they suggest that TRF may promote health through alternative mechanisms, such as enhanced circadian organization, behavioral rhythmicity, or tissue-specific metabolic adaptations. Consistent with this, 24-h EE and RER profiles revealed that TRF primarily reshaped temporal patterns of substrate utilization and EE, rather than altering overall 24-h mean values. Importantly, TRF had no detrimental impact on red blood cell indices, platelet counts, or immune cell abundance across aging, and the modest changes observed in 8-h TRF female mice were not associated with impaired oxygen-carrying capacity, supporting the long-term physiological safety of circadian aligned TRF.

By integrating measures of frailty, physiology, and behavior into a comprehensive healthspan index, we demonstrate that TRF significantly enhances healthspan in both sexes, with proportionally greater benefits in female mice relative to their lifespan. We also found that 12-h TRF strongly delayed the initial healthspan decline in female mice during middle-age, whereas its effect in male mice at the same threshold is minimal. However, this approach may oversimplify sex-specific differences, as male and female animals exhibit distinct patterns of health deficits that associate differently with longevity. A single healthspan scale as used here might not adequately reflect these complex, sex-dependent impacts on aging. Although female mice generally showed better health indices, we found they had shorter lifespans than male mice, highlighting a potential disconnect between this healthspan index and ultimate survival outcomes. It is also important to note, that sex differences observed in mice may not fully translate to humans, as differences in circadian rhythms, hormonal cycles and reproduction, and metabolic rate can alter the magnitude and timing of sex-specific responses to TRF^64–66^. Regardless, this sex-specific response in healthspan likely reflects a combination of biological factors. Estrogen is known to confer broad protective effects on health and aging^67^, which may explain the overall female advantage even under AL feeding, and it is known to feedback onto the circadian clock^68–70^. With recent literature showing that female humans possess a more robust circadian system^64^, this may underlie their enhanced responsiveness to circadian-based interventions. Recent studies have found that both female humans and rodents express a greater number of rhythmic genes, display higher amplitude rhythms, and are more sensitive to external cues^71–73^. As such, TRF may more effectively enhance rhythms at the molecular level to promote health in female mice^18,19^.

Importantly, we show that aligning feeding to the active phase under 12-h TRF does not extend lifespan. However, a narrower feeding window of 8-h TRF along with self-imposed CR conferred strong lifespan extension in male mice. These results contrast with previous studies showing that lifelong 20% CR, adjusted relative to age-related changes in AL feeding, extends lifespan by 40.6% in female and 24.4% in male C57BL/6 J mice^74^. Although our 8-h TRF mice maintained ~20% CR for nearly half of their lifespan, both male and female mice gradually lessened the degree of CR relative to AL feeding shortly before reaching median lifespan. Despite this, 8-h TRF male mice gained a 12% extension in median lifespan, indicating that even a lower degree of CR is still impactful. In contrast, female mice appear to require a sustained degree of CR ≥ 20% to see any lifespan benefits. Alternatively, the duration of fasting may be more crucial for female lifespan, as longer fasting periods with CR have enhanced lifespan effects in female mice^8,74^, but not in male^10^. The mechanisms underlying these sex-dependent differences in response to CR and TRF are not fully understood, especially between different strains of mice^5,74^, but are likely influenced by a complex interplay of factors such as differences in nutrient sensing and metabolic pathways, DNA repair, and the effects of sex hormones^39,64,75^.

Survival curves for AL mice unexpectedly showed an 18% increase in median survival for male mice compared to female, contrary to the common trend where female mammals typically live longer due to the genetic advantage of a backup X chromosome and the protective effects of estrogen^76,77^. However, in C57BL/6 J mice, lifespan outcomes are inconsistent possibly due to genetic drift within the isogenic line as well as environmental variations between experiments^77–79^. Although genetic factors may play a role, the shorter lifespan in female mice could stem from enhanced cold sensitivity and higher thermoregulatory energy demands^80–82^. The smaller-bodied female mice, needing more energy to maintain body temperature as evidenced by the pronounced increase in food intake with age, may have been adversely affected by the harsher individual housing conditions without nesting material. Although lower body temperatures can extend lifespan^83,84^, cold stress can also divert energy away from and impair metabolic, immune and reproductive functions^85,86^. Future studies will be needed to directly evaluate thermogenic pathways underlying these sex-specific effects, including gene expression in brown adipose tissue^45^. In addition, evaluating TRF and lifespan outcomes under thermoneutral housing conditions or with access to nesting material will help disentangle the relative roles of thermoregulation and feeding timing in shaping healthspan and longevity.

Health report monitoring and post-death histopathological analyses revealed significant differences between the sexes in the progression of age-related diseases, particularly in neoplasia. With the exception of lymphoma, female mice had significantly lower occurrences of neoplasms in the liver, lung, kidney and spleen which supports a growing body of data that female humans and mice have greater resistance to cancer^87–89^. This resistance is largely attributed to sex differences in immunity, mutational burden and DNA repair, as well as the protective role of X-linked tumor suppressor genes and their interaction with the p53 pathway in the female sex. Although mechanistic experiments are beyond the scope of this study, RNA-sequencing and proteomic analysis of the liver, the tissue most affected by neoplasia in male mice and least in female, could help to dissect the molecular mechanisms underlying these sex-specific differences in cancer development under TRF. These mechanisms are likely to include pathways regulating the cell cycle and cellular proliferation. Female mice in this cohort were instead most susceptible to kidney failure due to glomerulonephritis, similar to a previous study^90^. This condition has been linked to menopause in women, with its progression often accelerating after the decline in estrogen levels^91^. In mice, this disease can be further exacerbated in the absence of the estrogen receptor alpha gene^92^, emphasizing the protective role of estrogen signaling and uncovering renal disease as a potential biomarker of reproductive aging.

One limitation of this study was the inability of the automated feeders to retract or block a food pellet that goes uneaten beyond the restricted food intake window. This meant that often, a single pellet could be taken by the mouse, outside of its 12-h or 8-h TRF window. Additionally, some mice had the tendency to hoard pellets within their cage, leading again to food availability outside of the TRF window, a trait that has already been shown in aged mouse populations^42^. However, a single daytime pellet accounted for ≤6.34% of total caloric intake, and after subtracting hoarded food, mice consumed ≥90% of the pellets taken from the feeder during the correct feeding windows. These findings, along with evidence that TRF 5 out of 7 days per week regimens still improves metabolic health in mice^37^ and lifespan in flies^93^, suggest that moderate TRE adherence may still prolong healthspan and be compatible with modern human lifestyles.

Our study focused on TRF regimens initiated in early adulthood and maintained throughout life, leaving the efficacy of late-life implementation yet to be determined. Short-term studies indicate that TRF in older adults can reduce high-fat diet-induced inflammation and metabolic dysfunction, and studies with CR in aged mice still provided health and lifespan benefits^94–96^. However, these benefits provide diminishing returns, dependent on the age at which an intervention is started. Furthermore, as most of the observed benefits occurred through mid-life and the onset of old age, this raises the question of whether a lifelong intervention is necessary, or if stopping TRF before old age might be sufficient, or even preferable, to avoid potential late-life trade-offs. This possibility is supported by other models, where short-term TRF applied in flies earlier in life increases longevity and functional health even after returning to AL feeding^40,93,97^, and by some evidence that TRF in aged mice had negative impacts on lean mass and cardiovascular health^98^.

Beyond the life stage at which TRF is initiated, the specific timing of the feeding window relative to the internal circadian clock represents a critical variable in metabolic optimization. Here, the 8-h TRF window was intentionally positioned in the middle of the active period (ZT14-ZT22), instead of the onset (ZT12), consistent with prior mouse studies^28,99^, and human data suggesting metabolic benefits from delaying breakfast and/or advancing the final meal^100^. Thus, the enhanced healthspan and lifespan benefits observed with 8-h TRF may reflect not only feeding duration, but also a shift in the midpoint of daily caloric intake. Still, direct translation to humans is limited by species differences in metabolic rate and circadian timing, as fasting intervals in mice correspond to ~2.7- to 3.6-fold longer relative fasting windows in humans^66,101^.

The lack of direct cardiovascular assessments is also a limitation, particularly given the prevalence of age-related cardiovascular disease in mice. As long-term studies directly evaluating the cardiovascular effects of TRF/TRE remain limited^102,103^, future work should incorporate measures of cardiac structure, function and blood pressure to determine how timed feeding influences cardiovascular health across the lifespan during normal metabolic aging.

In conclusion, our study provides compelling evidence that the early initiation of circadian aligned TRF either via a 12-h or 8-h window significantly improves healthspan in both sexes, more so in female mice proportional to lifespan, and that 8-h TRF robustly extends lifespan in male mice. Although this still warrants further exploration in additional mouse strains and at varying ages of onset, taken together, this finding highlights the potential of TRF as a practical, nonpharmacological intervention to prolong the health quality of human lifespans.

## Methods

### Animals and housing conditions

The Institutional Animal Care and Use Committee of the University of Texas Southwestern Medical Center approved the animal protocol (APN 2015-100925), which has been renewed every 3 years (in 2018, 2021 and 2024).

A total of 264 male and 264 female C57BL/6 J mice (strain #000664) 6 weeks of age were ordered from Jackson Laboratories. We selected C57BL/6 J mice because they are widely used research model which exhibit robust circadian rhythms and well-known aging phenotypes^104,105^. Further, using a single, well-characterized strain maximized statistical power and lifespan resolution in our large cohort^106^. As done previously, starting at 2 months of age, the mice were individually housed in standard polycarbonate cages with stainless steel running wheels inside isolation cabinets under light:dark (LD) of 12:12 h and ambient building temperature 72–78°F, fed 300 mg pellets of purified diet (F0075, Bio-Serv) using automated feeders with water provided AL and cage-changed every 21 days^9,10,107^. Nesting material and igloos were excluded, besides shavings, to prevent wheel blockages.

The purified diet (F0075, Bio-Serv) is composed of 18.7% protein, 5.6% fat, 4.7% fiber and 59.1% carbohydrates, similar to regular mouse chows. Major ingredients include sucrose, dextrose, casein, corn oil, mineral mix, cellulose, corn syrup, calcium silicate, vitamin mix, magnesium stearate, choline bitartrate, DL-methionine, L-cystine, ascorbic acid, vitamin E acetate and tBHQ.

An additional cohort of C57BL/6 J animals (*n* = 48/feeding condition/sex) were included in this study to undergo more invasive assays. These mice were randomly selected (*n* = 8–12/group/age point) for analysis of metabolism in metabolic chambers, fasting blood glucose levels, glucose tolerance, biomarkers of inflammation and metabolism and hematology. These mice were not included in the longitudinal health measurements or the assessment of lifespan.

### TRF regimens

At 2 months of age, unmated mice were placed in individual cages as described above and fed AL for 8 weeks using the automated feeder system^9,10^. At 4 months of age, the mice were divided into three feeding groups for the remainder of the experiment: (i) 108 of each sex continued in AL as a control group, (ii) 78 of each sex in a 12-h TRF group where the automated feeder restricted food dispensing to the 12-h night (12-h TRF, ZT12-24) and (iii) 78 of each sex in an 8-h TRF group with food dispensing restricted to the middle 8 h of the night (8-h TRF, ZT14-22). These feeding windows were chosen as they have been previously validated and demonstrated to confer health benefits in mice^24,28^. In addition to the timing of the feeding window, the feeders were also programmed with a 10-min delay between pellet dispensing to reduce hoarding behavior. Regardless of the feeding group, all mice had access to a maximum of 22 pellets per day or 6.6 g of food. In this study, no group exceeded 6.6 g daily consumption. Previous studies have shown that C57BL/6 J mice consume 4–5 g of food per day^108^, so this amount of food was chosen to ensure mice were not calorically restricted.

### Exclusion of hoarded food

Mice exhibit a natural tendency to hoard food within the cage, especially later in life or when ill^42^. In all TRF groups, mice identified as hoarders were flagged, and hoarded food was removed from the cage on a weekly basis. For mice on veterinary health report, hoarded food was removed at their twice weekly full health check. The total amount of hoarded food was quantified by weight (g) at each 21-day cage change. For AL mice, hoarded food was allowed to remain in the cage until the scheduled cage change, at which point it was collected and quantified. In all groups, the total hoarded food weight was divided by 21 days to estimate an average daily hoarding amount, which was then subtracted from the average daily food retrieval recorded by the feeder.

### Daily monitoring of feeding and wheel-running activity

As before, the feeders were set and continuously recorded quantity and timing of food intake via ClockLab Chamber Control Software v3.401 (Actimetrics)^9,10^. The feeding data presented in Fig. 1b and Extended Data Fig. 2 represent food consumption with hoarding removed. All other feeding figures represent the quantity and pattern of pellet taking from the feeder, not excluding hoarded food. Wheel-running activity was also continuously recorded as before using an updated version of ClockLab Data Acquisition System v3.604 (Actimetrics).

### Body weight and composition measurements

The body weight of each mouse was measured every 21 days during cage change (morning, ZT4-7) throughout its lifespan. Body composition measurements of lean and fat mass (g) were assessed every 6 months during cage change (ZT4-7) using a 100H-EchoMRI Body Composition Analyzer (EchoMRI).

### Promethion metabolic chambers

These tests were performed in a follow-up cohort of mice. Mice were randomly selected.

At 19 months of age, mice received body composition measurements (EchoMRI) and then were individually housed in metabolic cages (Promethion, Sable Systems International) modified with automated feeders to maintain the same feeding regimens. These cages were housed in light-tight, temperature-controlled cabinets. Mice were acclimatized for 2 to 3 days at room temperature (25 °C) before recording for an additional 5 to 6 days at room temperature. Cabinet temperature was then raised to thermoneutrality (30 °C) for 5 days.

### Fasting glucose and glucose tolerance testing

These tests were performed in a follow-up cohort of mice. Mice were randomly selected from the cohort at each age point.

For fasting glucose, cages for all mice to be tested were changed out fresh the day before. Feeders for all groups were shut off at ZT21.5 to allow the mice 30 min to consume the last pellet dropped and officially begin their 12 h fast at ZT22. Mice were checked again at ZT0 to ensure no pellets remained in the cage or available in the pellet chute. At ZT10, a drop of blood was measured from the tail using an Accu-Check Glucose Meter.

For glucose tolerance testing, cages were changed fresh and mice fasted as described above. At ZT10, mice were injected with a 20% glucose solution at a dose of 0.1 ml per 25 g of body weight. A drop of blood from the tail was measured for glucose at the time of injection (0 min) and then at 15, 30, 45, 60 and 120 min after injection.

### Biomarkers of metabolism and inflammation

These markers were measured in a follow-up cohort of mice. Mice were randomly selected from the cohort at each age point.

Mice were switched to fresh cages and were fasted for 12 h as described above for fasting glucose. At ZT10, 70–100 µl blood was collected via submandibular bleed into K_2_ EDTA coated collection tubes (RAM Scientific Safe-T-Fill Capillary Blood Collection Systems) with cOmplete, Mini, EDTA-free Protease Inhibitor Cocktail (Roche) and DPP-IV (Millipore). Blood was immediately kept on ice and then spun down at 2,000*g* for 10 min at 4 °C. The upper layer of plasma was transferred to a new tube and stored at −80 °C until use.

Then, 20 to 25 µl plasma was measured for leptin, insulin, IL-1β, IL-6, IL-10, TNFα, MCP-1, and BDNF using a U-PLEX Adipokine Combo 1 (mouse) assay kit (Meso Scale Discovery; Cat #K15299K).

### Hematology

Measurements for red blood cells, white blood cells, and platelets were performed in a follow-up cohort of mice. Blood was collected at the same time from the same mice in the ‘Biomarkers of Metabolism and Inflammation’ section/experiment.

Mice were switched to fresh cages and were fasted for 12 h as described above for fasting glucose. At ZT10, 25 to 30 µl blood was collected via submandibular bleed into K_2_ EDTA coated collection tubes (RAM Scientific Safe-T-Fill Capillary Blood Collection Systems) with cOmplete, Mini, EDTA-free Protease Inhibitor Cocktail (Roche) and DPP-IV (Millipore). Then, 20 µl whole blood was measured in a Hemavet 950FS (Drew Scientific) at room temperature within 2 to 4 h of collection.

### Frailty scoring

Every 6 months, the mice were assessed for frailty or physical vulnerabilities according to 31 parameters of aging modified from Whitehead et al.^51^. These parameters include a range of physiological evaluations, scoring coat condition, ocular and auditory responses, physical and musculoskeletal state, respiration rate, body weight and temperature. Each assessment was scored as 0 (absent), 0.5 (mild) or 1 (severe). For BCSs, 0.5 was used for either a BCS of 2 or 4, and 1 was for a BCS of 1 or 5. Body weight and body temperature scores were determined by averaging the group measurements and then determining how many standard deviations each individual mouse was from their age-, sex- and feeding-matched group mean. If <1 s.d., mouse scored as 0 for that parameter, 1–1.99 s.d. = 0.25, 2–2.99 s.d. = 0.5, 3–3.99 = 0.75, and >4 s.d. = 1. Scores were totaled to calculate an overall index score. Two scorers assessed each mouse and were blinded to the identity of the mouse’s feeding group.

### Health monitoring and survival study

Mice were physically health checked every 21 days during cage change and visually inspected every 10 days during refilling of the automated feeders. Using the feeding and wheel monitoring software, mice were also checked virtually every day and only physically health checked if feeding fell to less than five pellets per day and/or wheel running fell below set thresholds (1.14 counts per minute within 24 h, reduced by 10% every 6 months as the mice age) as performed previously^10^.

If any mouse was found to have a mild to moderate health ailment (for example, dermatitis, external tumor, abdominal mass, head tilt) or a BCS < 3 (scored 1–5, with 1 being emaciated, 3 ideal condition, and 5 obese), it was placed on veterinary health report. These records were also used to generate Fig. 4d–f. Mice on health report were physically health checked weekly by the UT Southwestern Animal Resource Facility veterinary staff and twice a week by our own laboratory staff. To obtain accurate but humane lifespan data, mice were monitored carefully for euthanasia criteria that would indicate imminent death (moribund) or unacceptable levels of pain (analgesics were not used to avoid confounding lifespan results). Criteria were determined in conjunction with UT Southwestern Animal Resource Facility veterinarians according to AAALAC guidelines and include nonresponsive to touch, hypothermic or cold to touch, slow or labored breathing, failure or inability to eat/drink after moist chow is given, a BCS of 1, body weight loss of >20% from baseline or between cage changes, a broken/fractured/dislocated limb, head tilt that is resulting in an animal being unable to maintain sternal body position that would affect their ability to consume food and water, severe eye protrusion/infection, severe dermatitis, grade 3 rectal or penile prolapse, urinary obstruction or a subcutaneous tumor >2 cm.

The number and date of those euthanized or found dead across the feeding groups and sexes were recorded and used to generate Kaplan-Meier survival curves. A total of 35 animals (35/528) were censored from the survival curve if death occurred earlier than 6 months or due to non-aging-related injuries or death, including 9 AL female, 6 12-h TRF female, 6 8-h TRF female, 9 AL male and 5 8-h TRF male mice.

### Necropsy and histopathology

Mice euthanized or found dead were submitted for gross necropsy and blinded histopathological analysis was performed, as done previously^10^. Less than 10% of the animals submitted were too autolyzed to analyze. Disease types were tallied together by tissue type, sex and feeding group.

### Healthspan index

Throughout the experiment, we recorded 13 health measures, including wheel-running activity to determine overall activity level and diurnal amplitude, food consumption to determine overall level and diurnal amplitude, 90% feeding duration, maximum fasting times, body weight, composition of lean and fat mass as a percentage of body weight, BCS, frailty index and the onset and duration of veterinary health reports.

Healthspan index was calculated by summing the calculated scaled values of each of the thirteen health measures. In the case of BCS, an absolute difference from BCS = 3 was calculated. For body fat percentage, an absolute difference in value from 24.45% was calculated. The value 24.45% was used based on previous literature showing the average body fat percentage across multiple healthy mouse strains at the age of 16 weeks^109^. All measures were then normalized to a mean of 0 and a s.d. of 1, within each measure. Certain values were then inverted, given their context, to have positive numbers representing a healthier state, and negative values representing an unhealthy state. For example, ‘days on health report’ is inverted, as higher values would be less healthy, and lower values are healthier. All normalized values were then summed together at 21-day intervals.

### FAMY

FAMY was calculated using the lifetime of frailty scores collected for each mouse and as described in Lamming 2024^55^.

### Statistics and reproducibility

For a minimum detection of 10% life extension with 80% power, a minimum of 96 animals were needed in the control group and 72 in each of the two TRF groups^106^. Additional mice were included in each group to account for any that may not adapt to the feeder/diet or for non-aging-related deaths. Each individual mouse was treated as a biological replicate (experimental unit). All health measurements represent repeated measures from the same animals over time, with the exception of data shown in Supplementary Figs. 1–7, which were collected from a separate cohort using different mice at each age point. Sample sizes (*n*) for each experiment are indicated in the corresponding figure legends. For both the longevity and follow-up cohort, during transfer from group housing (five mice per cage) to individually housed automated feeder cages, mice were assigned to feeding conditions using a systematic allocation approach. Specifically, animals were assigned sequentially in a rotating manner to each group (AL, 12-h TRF, 8-h TRF) as they were moved. Following assignment, groups were verified to have similar mean body weights and normally distributed body weight distributions prior to initiation of feeding regimens. For the follow-up cohort of mice used in Supplementary Figs. 1–7, mice were randomly sampled from each feeding group and sex for experiments.

As performed previously, all feeding and wheel-running activity was collected using Chamber Control and ClockLab, respectively (Actimetrics)^10^. Each point on the longitudinal profiles represents a 21-day mean. 24-h profiles were binned by hour, with each age point reflecting a 14-day mean (taken from the middle of each 21-day cage cycle to avoid behavioral changes due to cage change stress). For each mouse, each 14-day bin was then analyzed by one-dimensional discrete FFT using the mixed-radix algorithm for real-valued input (SciPy v1.12.0; scipy.fft.rfft)^110^. The Blackman window function is applied to the data prior to FFT analysis to inhibit spectral leakage^111^. The power of each frequency component is then normalized, with the total normalized power of all frequency components equaling 100. The normalized power from the 24-h frequency component is then taken as the diurnal amplitude of either food intake behavior or running wheel activity. This value represents the percentage of contribution of the 24-h frequency component, to the entire 14-day behavioral period. For example, if the diurnal amplitude for food intake is 21, it indicates that 21% of the observed variation in food intake can be directly attributed to the animal’s consistent 24-h daily cycle. Group-wise statistics were then performed to determine the overarching impact of each intervention, as described below.

For the calculation of the 90% feeding window, food intake data was similarly partitioned into 3-week intervals, with each sampling point consisting of a 14-day window. Only windows containing a minimum of 84 feeding events (averaging 6 events per day, or ~1.8 g of intake) were included in the final density analysis to ensure robust statistical estimation. Because time of day is a periodic variable, we used a von Mises distribution (the circular analogue of the Normal distribution) to estimate the probability density of feeding activity. We utilized the vonmises.pdf method (SciPy v1.12.0), to compute the circular distribution, where the concentration parameter κ, was defined by a fixed bandwidth of 0.04363 radians, representing approximately 10 min of smoothing. To define the primary feeding window, we identified the shortest continuous arc on the circle containing 90% of the total probability mass. This method accounts for feeding windows that cross over the ZT24/ZT0 boundary.

Cumulative health report and survival curves were analyzed using Log-rank Mantel Cox and Fisher’s exact test^112^, health report type and necropsy report disease type percentages analyzed by contingency tables and Fisher’s exact test and all other plots analyzed using two-way ANOVA or type III ANOVA with Tukey’s or Holm’s post-hoc analyses. All statistical tests were two sided. Normality was confirmed using Shapiro–Wilk tests and/or visual inspection of Q-Q plots of ANOVA residuals. Due to sufficiently large sample sizes per group, parametric analyses were used even when some data sets deviated from normality, consistent with the Central Limit Theorem. Data plots were generated and statistically analyzed by Prism 10 (GraphPad Software), Python and R software, using custom-written scripts and standard libraries.

### Reporting summary

Further information on research design is available in the Nature Portfolio Reporting Summary linked to this article.

## Supplementary information

Supplementary Information Supplementary Figures 1–7.

Reporting Summary

Peer Review File

Supplementary Table 1 Descriptive statistics for all ANOVA tests performed across all main figures, extended data figures, and supplementary figures.

Supplementary Table 2 Details of survival percentiles by day, and contingency tables and Fisher’s exact test for maximum lifespan detection for Fig. 5 a.

Supplementary Table 3 Full table of histopathology results with disease tallies and disease percentages per group.

Supplementary Table 4 Contingency tables and Fisher’s exact test for differences in occurrence of diseases listed in Fig. 5b.

## Data Availability

Source data have been deposited 10.6084/m9.figshare.31894711 (ref. ^113^), and all data supporting the findings of the study are available from the corresponding authors upon request.

## Code availability

Custom codes used to analyze the source data can also be found at 10.6084/m9.figshare.31894711 and is provided as a single file containing sectioned analysis code in both .Rmd and .md formats^113^.
