# Supplementary material for: Time-restricted feeding extends healthspan in both sexes and lifespan in male C57BL/6 J mice
Source: Nat Aging. 2026 Jun 2;6(6):1227–43. doi: 10.1038/s43587-026-01129-8 (PMC13303084; doi:10.1038/s43587-026-01129-8)
Supplement: Supplementary file 1 — Supplementary Figures 1–7. [file 43587_2026_1129_MOESM1_ESM.pdf]

nature aging

Supplementary information https://doi.org/10.1038/s43587-026-01129-8

Time-restricted feeding extends healthspan in both sexes and lifespan in male C57BL/6 J mice

In the format provided by the authors and unedited

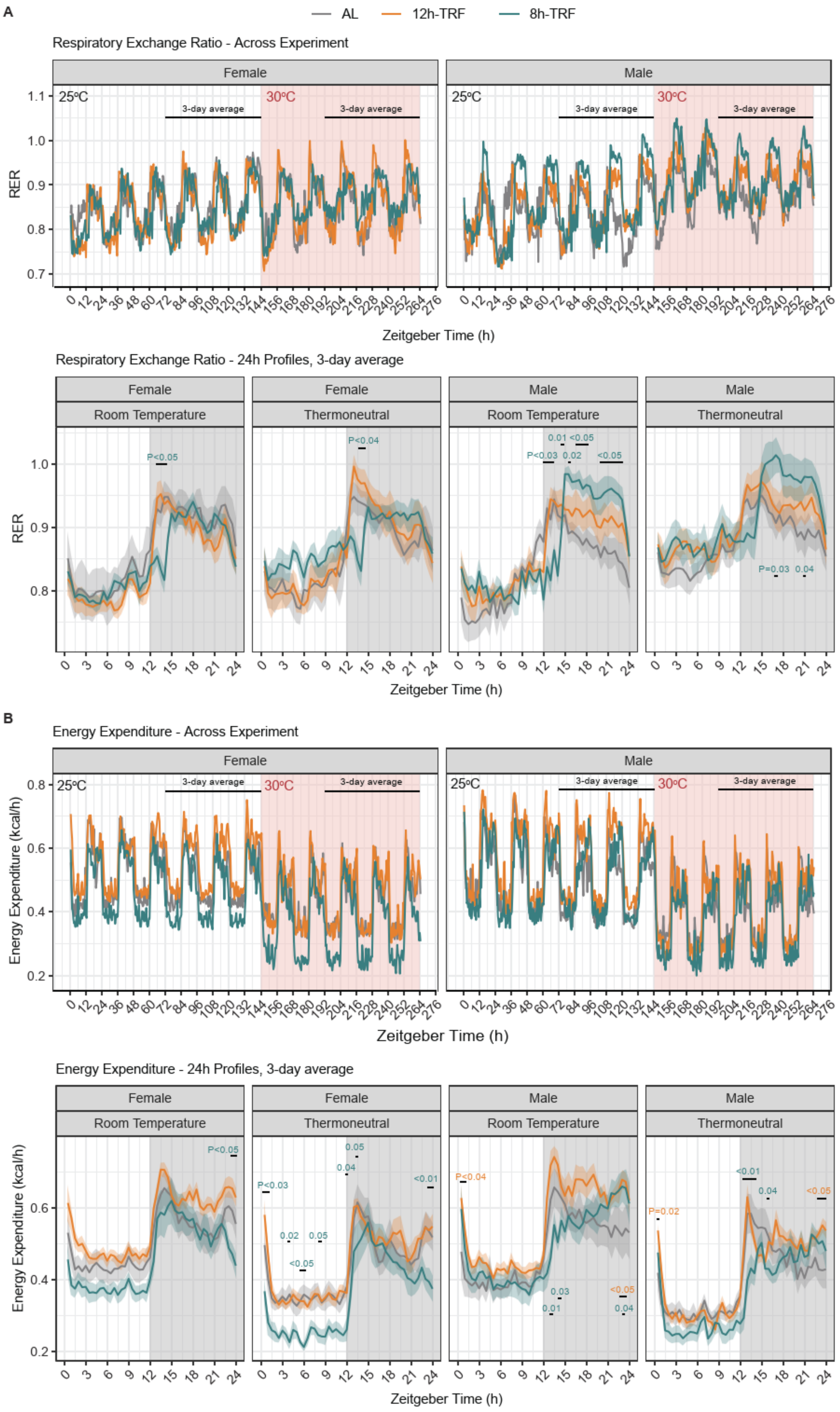

Supplementary Figure 1: Measures of metabolism at 19 months of age in a follow-up cohort

of mice: (**A**) (*Top*) Respiratory exchange ratios (RER; VCO2/VO2) in female and male mice throughout a 11-day recording in Promethion metabolic chambers. 6 days recorded at room temperature (25ºC; white shaded area), and 5 days were recorded at thermoneutrality (30ºC; red shaded area). (*Bottom*) 24-h profiles of RER averaged from the last 3 days of recording in each temperature. 12 h light (white shaded area), 12 h dark (gray shaded area). Means ± SEM (shading) presented in 30-minute bins. (**B**) (*Top*) Energy expenditure (EE; kcal/h) in female and male mice throughout the 11-day recording. 6 days recorded at room temperature (25ºC; white shaded area), and 5 days were recorded at thermoneutrality (30ºC; red shaded area). Measurements are presented in 30-minute bins. (*Bottom*) 24-h profiles of EE from the last 3 days of recording in each temperature. 12 h light (white shaded area), 12 h dark (gray shaded area). Means ± SEM (shading) presented in 30-minute bins. A and B: Significant differences ($P \leq 0.05$) in TRF vs AL (colored P-values; orange:12-h TRF, teal:8-h TRF) as determined by type III ANOVA (Wald χ^2), and Holm's post-hoc. Female: AL, N=6. 12-h TRF, N=7. 8-h TRF, N=7. Male: AL, N=7. 12-h TRF, N=8. 8-h TRF, N=6.

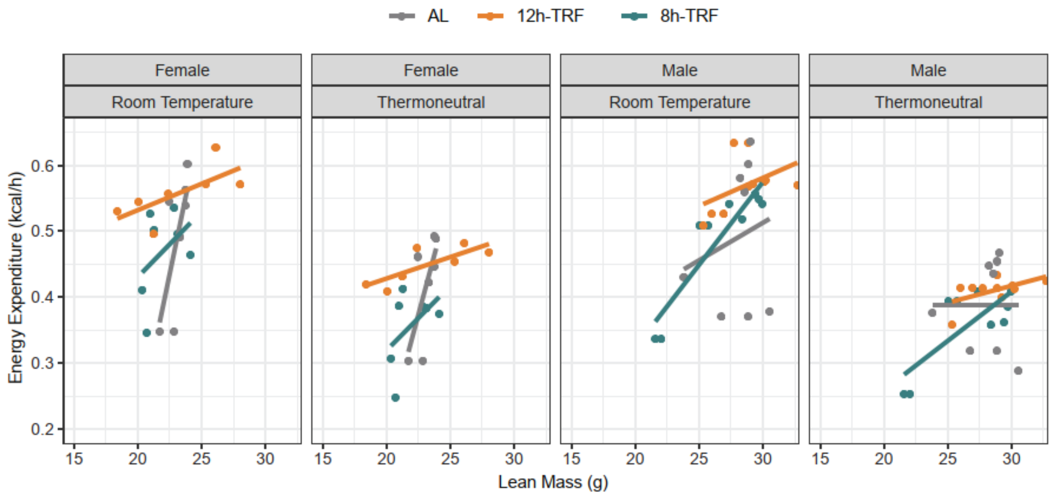

Female Room Temperature						
1. INTERACTION MODEL (Slope Consistency)						
	Df	Sum Sq	Mean Sq	F value	Pr(>F)	signif
Lean	1	0.02322	0.02322	5.97928	0.02729	*
feeding	2	0.02204	0.01102	2.83776	0.09011	ns
Lean:feeding	2	0.02754	0.01377	3.54642	0.0548	ns
Residuals	15	0.05825	0.00388			
2. ANCOVA MODEL (Type III SS)						
	Sum Sq	Df	F value	Pr(>F)	signif	
(Intercept)	0.00554	1	1.09845	0.30928	ns	
Lean	0.01599	1	3.16844	0.09295	ns	
feeding	0.02204	2	2.1836	0.14321	ns	
Residuals	0.08579	17				

Female Thermoneutrality						
1. INTERACTION MODEL (Slope Consistency)						
	Df	Sum Sq	Mean Sq	F value	Pr(>F)	signif
Lean	1	0.02085	0.02085	8.61881	0.01022	*
feeding	2	0.02061	0.0103	4.25862	0.0343	*
Lean:feeding	2	0.01696	0.00848	3.50538	0.05635	ns
Residuals	15	0.03629	0.00242			
2. ANCOVA MODEL (Type III SS)						
	Sum Sq	Df	F value	Pr(>F)	signif	
(Intercept)	0.00416	1	1.32941	0.26487	ns	
Lean	0.01125	1	3.59171	0.0752	ns	
feeding	0.02061	2	3.28915	0.06201	ns	
Residuals	0.05325	17				

Male Room Temperature						
1. INTERACTION MODEL (Slope Consistency)						
	Df	Sum Sq	Mean Sq	F value	Pr(>F)	signif
Lean	1	0.06713	0.06713	12.7309	0.00182	**
feeding	2	0.02161	0.01081	2.04916	0.15383	ns
Lean:feedin	2	0.00929	0.00464	0.88045	0.42935	ns
Residuals	21	0.11074	0.00527			
2. ANCOVA MODEL (Type III SS)						
	Sum Sq	Df	F value	Pr(>F)	signif	
(Intercept)	2.8E-05	1	0.00528	0.94269	ns	
Lean	0.04917	1	9.42346	0.00542	**	
feeding	0.02161	2	2.07069	0.14897	ns	
Residuals	0.12002	23				

Male Thermoneutrality						
1. INTERACTION MODEL (Slope Consistency)						
	Df	Sum Sq	Mean Sq	F value	Pr(>F)	signif
pre_lean	1	0.02215	0.02215	9.1224	0.00651	**
feeding	2	0.00477	0.00239	0.98324	0.39067	ns
pre_lean:fee	2	0.00572	0.00286	1.17758	0.32755	ns
Residuals	21	0.05099	0.00243			
2. ANCOVA MODEL (Type III SS)						
	Sum Sq	Df	F value	Pr(>F)	signif	
(Intercept)	0.00285	1	1.1577	0.2931	ns	
pre_lean	0.01371	1	5.55981	0.02726	*	
feeding	0.00477	2	0.96829	0.39468	ns	
Residuals	0.05671	23				

Supplementary Figure 2: ANCOVA of TRF effects on energy expenditure: 24-h mean EE plotted vs lean mass for female and male mice. Homogeneity of regression slopes was confirmed for all groups (test of Feeding × Lean Mass interaction, all $p > 0.05$, ns), validating the use of ANCOVA to test for significance of intercepts. Female: AL, N=6. 12-h TRF, N=7. 8-h TRF, N=7. Male: AL, N=7. 12-h TRF, N=8. 8-h TRF, N=6.

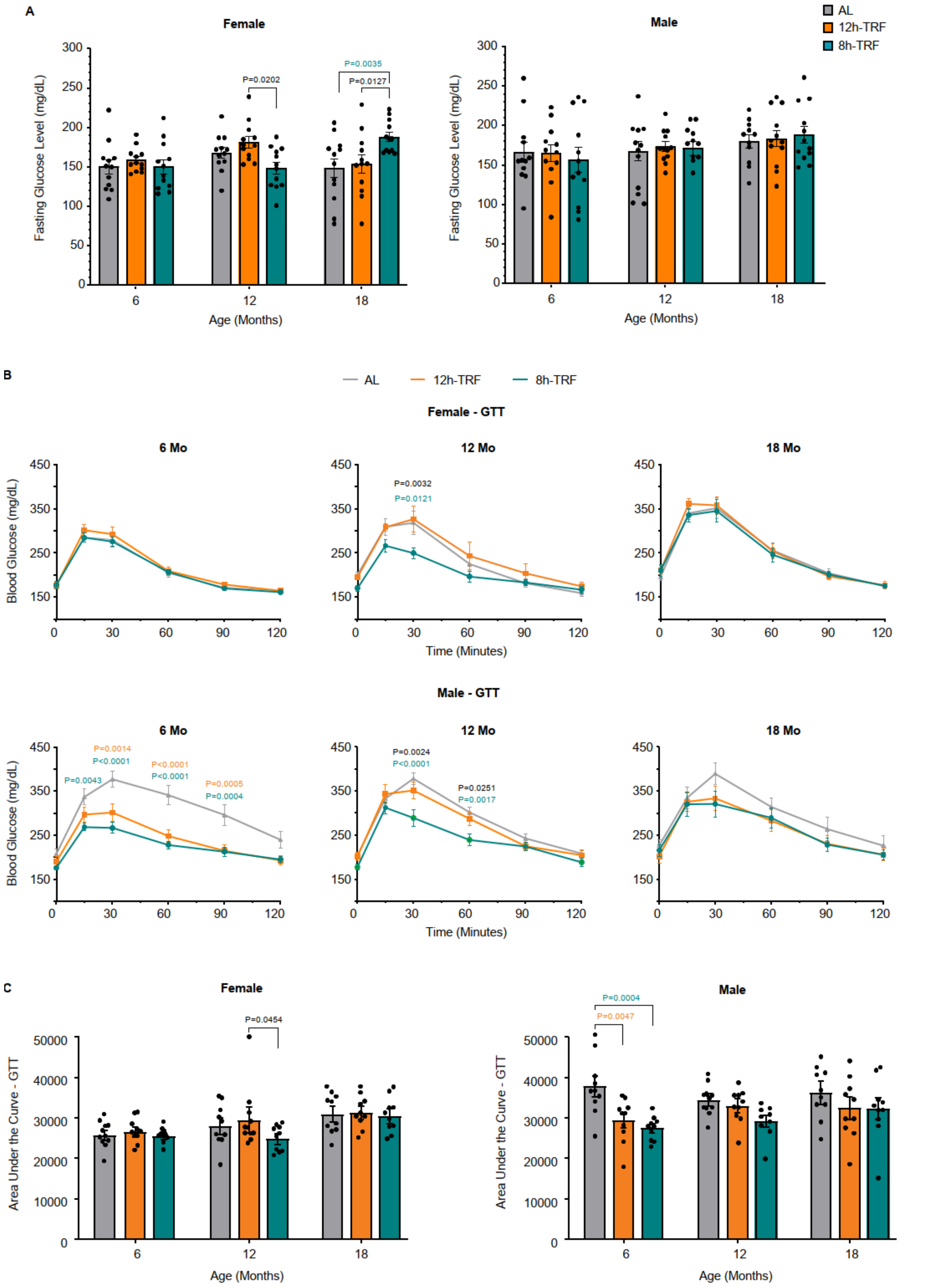

Supplementary Figure 3: Fasting glucose levels and glucose tolerance tests in follow-up

mouse cohort: In female and male mice: (**A**) Bar graphs of 12-h fasting glucose levels (mg/dL) collected at zeitgeber time (ZT) 10, Female: N=12/group/age, Male: AL 6 months N=12, 12 and 18 months N=11, 12-h/8-h TRF N=12/age, (**B**) glucose tolerance tests (GTT) performed starting at ZT10, and (**C**) area under the curve (AUC) for the GTTs tested at 6, 12, and 18 months of age. Panels B and C: Female 6, 12, and 18 months: AL, N=11, 10, 10. 12-h TRF, N=10, 11, 10. 8-h TRF, N=10, 10, 10. Male 6, 12, and 18 months: AL, N=10, 11, 10. 12-h TRF, N=10, 10, 10. 8-h TRF, N=10, 9, 10. All panels: Means ± SEM (bars). Significant differences in TRF vs AL (colored P-values; orange:12-h TRF, teal:8-h TRF) and 12-h vs 8-h TRF (black colored P-values) as determined by two-way ANOVA and Tukey's post-hoc.

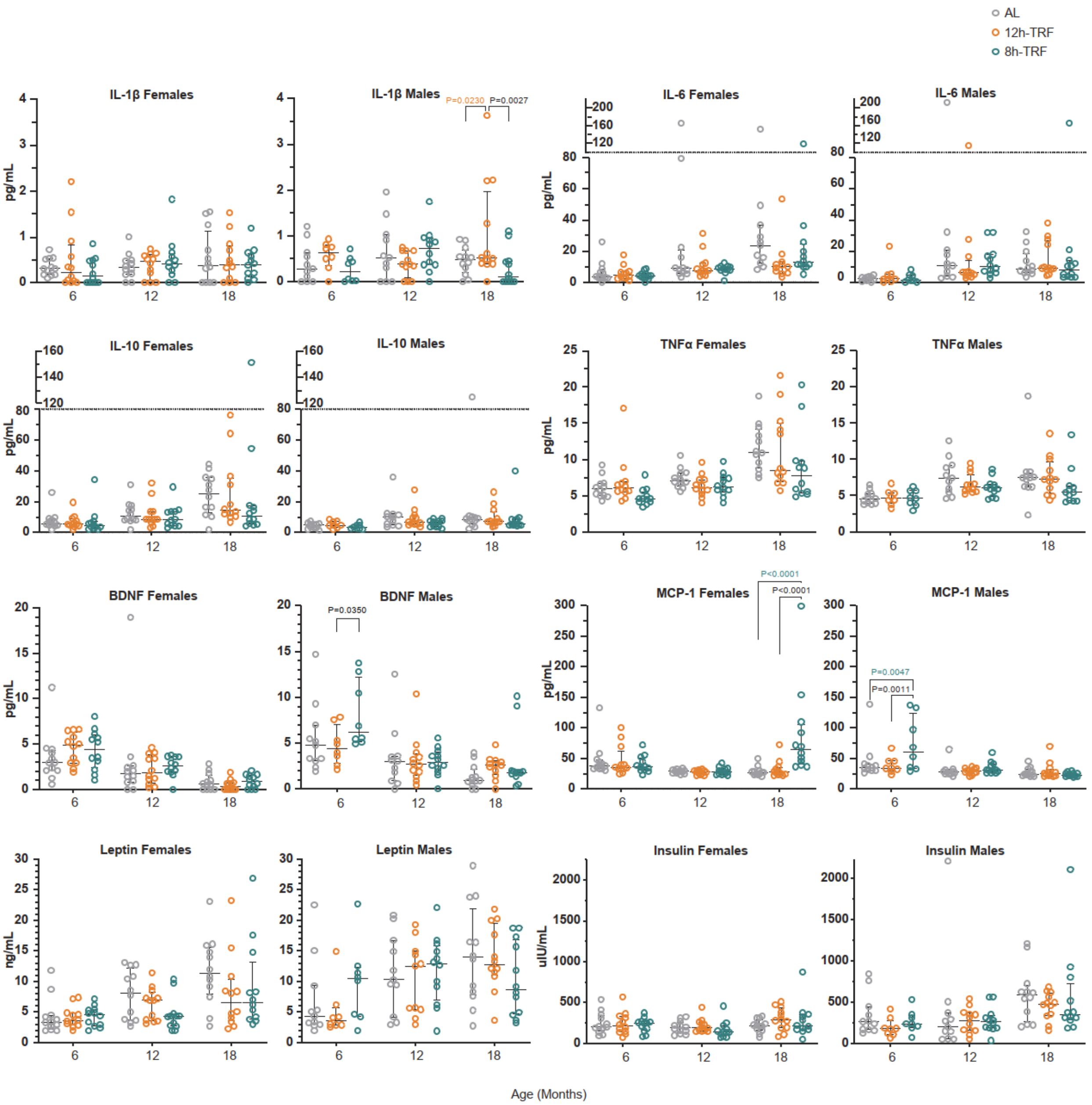

Supplementary Figure 4: Circulating levels of adipokines from blood plasma in follow-up mouse cohort. In female and male mice, dot plots of circulating levels of interleukin (IL)-1β, IL-6, IL-10, tumor necrosis factor α (TNFα), brain-derived neurotrophic factor (BDNF), monocyte chemoattractant protein (MCP)-1, leptin, and insulin. Plasma was collected at ZT10 from 12-h fasted blood at 6, 12, and 18 months of age. Median (wide middle line) with interquartile range (narrow top and bottom lines). Significant differences in TRF vs AL (colored P-values; orange:12-h TRF, teal:8-h TRF) and 12-h vs 8-h TRF (black colored P-values) as determined by two-way ANOVA and Tukey's post-hoc. Female 6, 12, and 18 months: AL, N=12, 12, 12. 12-h TRF, N=12, 12, 12. 8-h TRF, N=12, 12, 12 for all analytes with exception of IL-6 at 18 months where AL and 12-h TRF were N=11. Male 6, 12, and 18 months: AL, N=11, 11, 12. 12-h TRF, N=8, 12, 12. 8-h TRF, N=8, 12, 12 for all analytes.

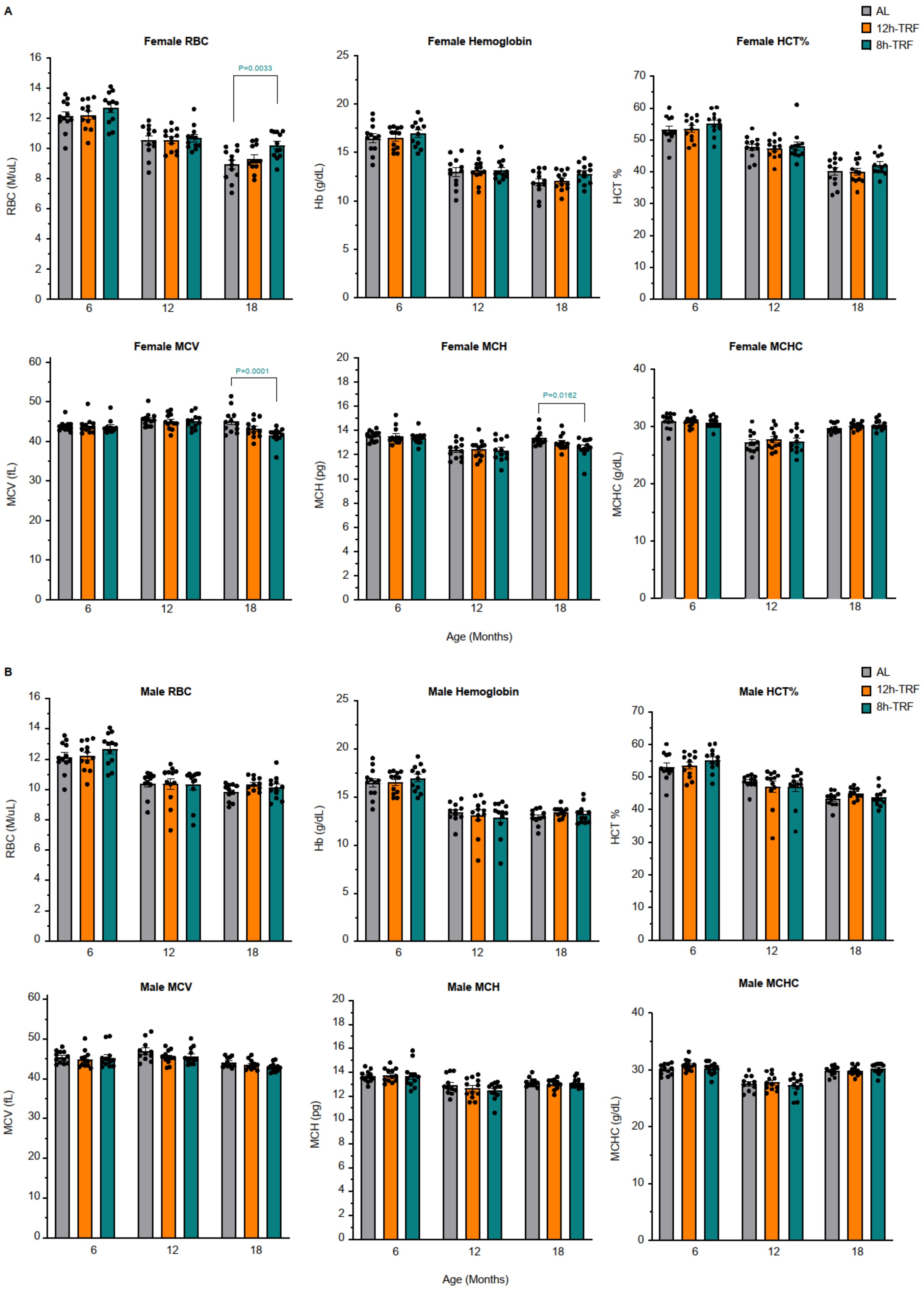

Supplementary Figure 5: Red blood cell measures with age in follow-up mouse cohort. (A)

In female and (**B**) male mice, bar graphs of red blood cell (RBC) counts (M/µL), hemoglobin levels (g/dL), hematocrit (HCT, percentage of RBCs in total blood volume) (%), mean corpuscular volume (MCV, average size of RBCs) (fL), mean corpuscular hemoglobin (MCH, average amount of hemoglobin per RBC) (pg), and mean corpuscular hemoglobin concentration (MCHC, average concentration of hemoglobin within the volume of the RBC) (g/dL). Measured at ZT10 from 12-h fasted blood at 6, 12, and 18 months of age. Mean ± SEM (bars). Significant differences in TRF vs AL (colored P-values; orange:12-h TRF, teal:8-h TRF) and 12-h vs 8-h TRF (black colored P-values) as determined by two-way ANOVA and Tukey's post-hoc**.** Female: N=12/group/age. Male 6, 12, and 18 months: AL, N=12, 11, 11. 12-h TRF, N=12, 12, 11. 8-h TRF, N=12, 12, 12.

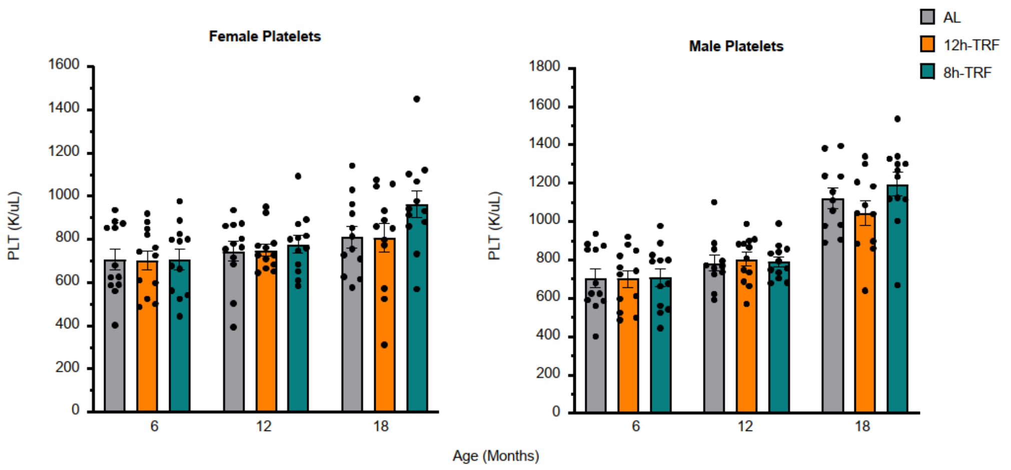

Supplementary Figure 6: Platelet counts with age in follow-up mouse cohort. In female and male mice, platelet counts (K/µL). Measured at ZT10 from 12-h fasted blood at 6, 12, and 18 months of age. Mean ± SEM (bars). Two-way ANOVA, Tukey's post-hoc. Female: N=12/group/age. Male 6, 12, and 18 months: AL, N=12, 11, 11. 12-h TRF, N=12, 12, 11. 8-h TRF, N=12, 12, 12.

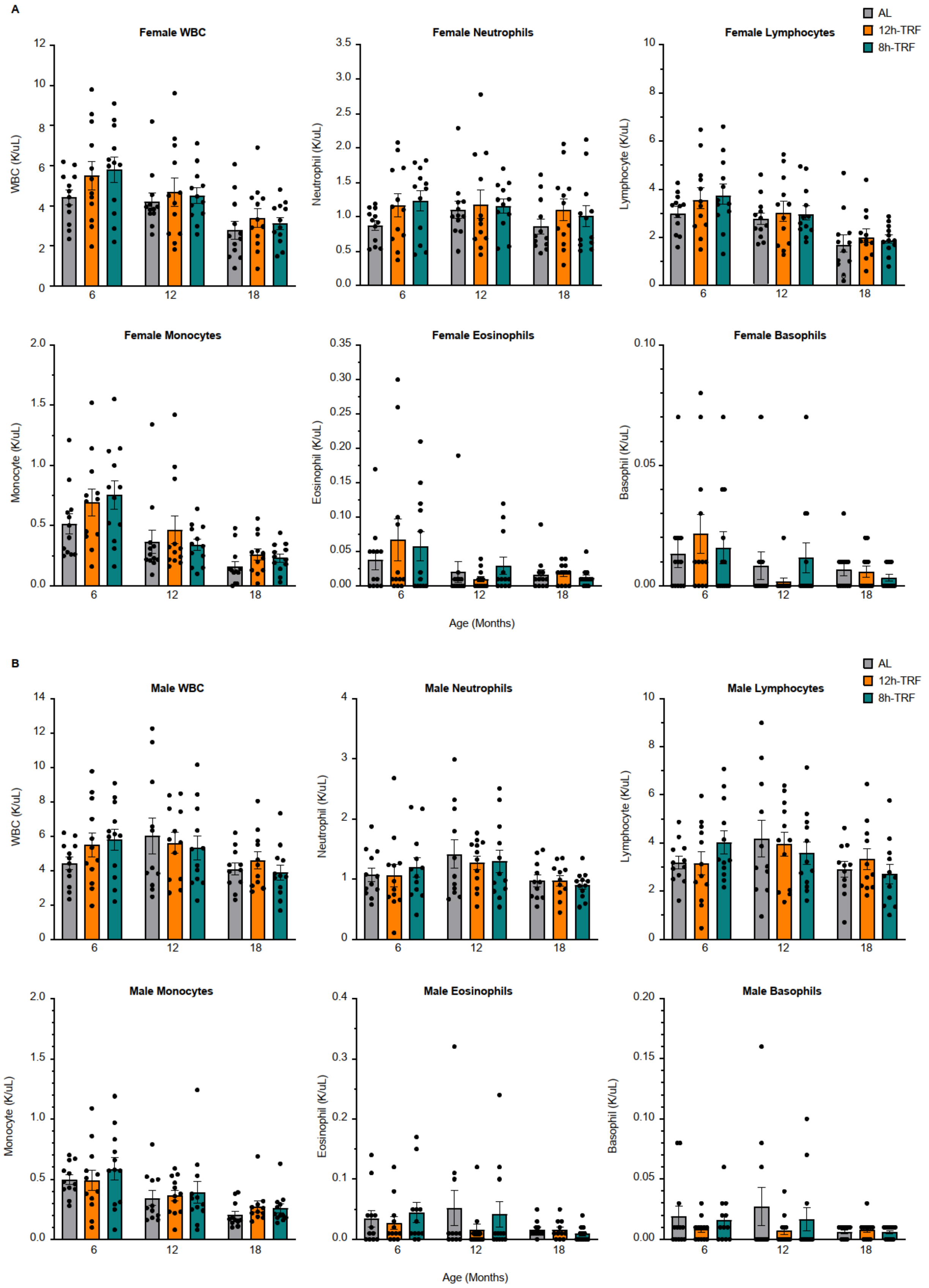

Supplementary Figure 7: White blood cells measures with age in follow up mouse cohort.

(**A**) In female and (**B**) male mice, bar graphs of total white blood cell (WBC) counts (K/μL) and counts for each WBC sub-type including neutrophils, lymphocytes, monocytes, eosinophils, and basophils. Measured at ZT10 from 12-h fasted blood at 6, 12, and 18 months of age. Mean ± SEM (bars). Two-way ANOVA, Tukey's post-hoc. Female: N=12/group/age. Male 6, 12, and 18 months: AL, N=12, 11, 11. 12-h TRF, N=12, 12, 11. 8-h TRF, N=12, 12, 12.
