# Supplementary material for: Time-restricted feeding extends healthspan in both sexes and lifespan in male C57BL/6 J mice
Source: Nat Aging. 2026 Jun 2;6(6):1227–43. doi: 10.1038/s43587-026-01129-8 (PMC13303084; doi:10.1038/s43587-026-01129-8)
Supplement: Supplementary file 2 — Reporting Summary [file 43587_2026_1129_MOESM2_ESM.pdf]

Corresponding author(s): Victoria A. Acosta- Rodríguez; Carla B. Green; Joseph S. Takahashi

Last updated by author(s): Apr 13, 2026

Reporting Summary

Nature Portfolio wishes to improve the reproducibility of the work that we publish. This form provides structure for consistency and transparency in reporting. For further information on Nature Portfolio policies, see our Editorial Policies and the Editorial Policy Checklist.

Statistics

For all statistical analyses, confirm that the following items are present in the figure legend, table legend, main text, or Methods section.

n/a	Confirmed	
☐	☒	The exact sample size (*n*) for each experimental group/condition, given as a discrete number and unit of measurement
☐	☒	A statement on whether measurements were taken from distinct samples or whether the same sample was measured repeatedly
☐	☒	The statistical test(s) used AND whether they are one- or two-sided<br>*Only common tests should be described solely by name; describe more complex techniques in the Methods section.*
☐	☒	A description of all covariates tested
☐	☒	A description of any assumptions or corrections, such as tests of normality and adjustment for multiple comparisons
☐	☒	A full description of the statistical parameters including central tendency (e.g. means) or other basic estimates (e.g. regression coefficient) AND variation (e.g. standard deviation) or associated estimates of uncertainty (e.g. confidence intervals)
☐	☒	For null hypothesis testing, the test statistic (e.g. *F*, *t*, *r*) with confidence intervals, effect sizes, degrees of freedom and *P* value noted<br>*Give P values as exact values whenever suitable.*
☒	☐	For Bayesian analysis, information on the choice of priors and Markov chain Monte Carlo settings
☒	☐	For hierarchical and complex designs, identification of the appropriate level for tests and full reporting of outcomes
☐	☒	Estimates of effect sizes (e.g. Cohen's *d*, Pearson's *r*), indicating how they were calculated

Our web collection on statistics for biologists contains articles on many of the points above.

Software and code

Policy information about availability of computer code

Data collection	Actimetrics ClockLab Software, Actimetrics ChamberControl Software
Data analysis	Graphpad Prism Software, ClockLab Anaylsis, custom R and Python scripts

For manuscripts utilizing custom algorithms or software that are central to the research but not yet described in published literature, software must be made available to editors and reviewers. We strongly encourage code deposition in a community repository (e.g. GitHub). See the Nature Portfolio guidelines for submitting code & software for further information.

Data

Policy information about availability of data

All manuscripts must include a data availability statement. This statement should provide the following information, where applicable:

- Accession codes, unique identifiers, or web links for publicly available datasets
- A description of any restrictions on data availability
- For clinical datasets or third party data, please ensure that the statement adheres to our policy

The primary experimental data supporting the findings of this study have been deposited in a Figshare repository under accession code/DOI 10.6084/m9.figshare.31894711. The R and Python code used for data analysis and processing are publicly available at the same Figshare repository. Extended data and any other remaining data that support the findings of this study are available from the corresponding author upon reasonable request.

Research involving human participants, their data, or biological material

Policy information about studies with human participants or human data. See also policy information about sex, gender (identity/presentation), and sexual orientation and race, ethnicity and racism.

Reporting on sex and gender	N/A
Reporting on race, ethnicity, or other socially relevant groupings	N/A
Population characteristics	N/A
Recruitment	N/A
Ethics oversight	N/A

Note that full information on the approval of the study protocol must also be provided in the manuscript.

Field-specific reporting

Please select the one below that is the best fit for your research. If you are not sure, read the appropriate sections before making your selection.

☒ Life sciences ☐ Behavioural & social sciences ☐ Ecological, evolutionary & environmental sciences

For a reference copy of the document with all sections, see nature.com/documents/nr-reporting-summary-flat.pdf

Life sciences study design

All studies must disclose on these points even when the disclosure is negative.

Sample size	For a minimum detection of 10% life extension with 80% power a minimum of 96 animals were needed in the control group and 72 in each of the two TRF groups (Miller et al., Aging Cell 2019). 108 of each sex continued in AL as a control group, 78 of each sex in a 12-hour TRF group where the automated feeder restricted food dispensing to the 12-hour night (12h-TRF, ZT12-24), and 78 of each sex in an 8-hour TRF group with food dispensing restricted to the middle 8 hours of the night (8h-TRF, ZT14-22). Extra mice were included in each group to account for any that may not adapt to the feeder/diet or for non-aging-related deaths.
Data exclusions	A total of 35 animals were censored from the survival curve if death occurred earlier than 6 months or due to non-aging related injuries or death: 9 AL female, 6 12h-TRF female, 6 8h-TRF female, 9 AL male and 5 8h-TRF male mice.
Replication	Due to the long-term and resource-intensive nature of lifespan studies, experiments were not independently repeated. Instead, reproducibility was supported by the use of large cohort sizes, longitudinal study design, and consistent effects observed across multiple health and behavioral measures repeated across each mouse's lifespan.
Randomization	Mice were randomly assigned to one of the three feeding conditions via systematic allocation during transfer from group housing to individual automated feeder cages. Each group was verified to have equal means and normally distributed body weights prior to starting the feeding regimens.<br><br>For the follow-up cohort of mice used to test metabolism, glucose homeostasis, circulating blood markers, and hematology, mice were randomly selected within this cohort at each age point for one of the above tests.
Blinding	Blinding was used during frailty scoring. Scorers were blinded to the identity and experimental condition of the mouse.<br>Dr. Mary Wight-Carter was also blinded to the TRF regimen of each mouse for the histopathological analysis.

Reporting for specific materials, systems and methods

We require information from authors about some types of materials, experimental systems and methods used in many studies. Here, indicate whether each material, system or method listed is relevant to your study. If you are not sure if a list item applies to your research, read the appropriate section before selecting a response.

Materials & experimental systems

n/a	Involved in the study
☐	☐ Antibodies
☐	☐ Eukaryotic cell lines
☐	☐ Palaeontology and archaeology
☐	☒ Animals and other organisms
☐	☐ Clinical data
☐	☐ Dual use research of concern
☐	☐ Plants

Methods

n/a	Involved in the study
☐	☐ ChIP-seq
☐	☐ Flow cytometry
☐	☐ MRI-based neuroimaging

Antibodies

Antibodies used: For analyzing circulating inflammatory and metabolic markers, antibodies used were supplied with the U-PLEX Adipokine Combo 1 (mouse) assay kit (Meso Scale Discovery; Cat #: K15299K).

Validation: Antibody combination validated by Meso Scale Discovery for commercial sale.

Eukaryotic cell lines

Policy information about cell lines and Sex and Gender in Research

Cell line source(s): N/A

Authentication: N/A

Mycoplasma contamination: N/A

Commonly misidentified lines (See ICLAC register): N/A

Palaeontology and Archaeology

Specimen provenance: N/A

Specimen deposition: N/A

Dating methods: N/A

☐ Tick this box to confirm that the raw and calibrated dates are available in the paper or in Supplementary Information.

Ethics oversight: N/A

Note that full information on the approval of the study protocol must also be provided in the manuscript.

Animals and other research organisms

Policy information about studies involving animals; ARRIVE guidelines recommended for reporting animal research, and Sex and Gender in Research

Laboratory animals: Animals: C57BL/6J Mice (JAX Strain #:000664), Males and Females, Starting at 2 months of age to end of life.

Housing Conditions: starting at 2 months of age the mice were: (i) individually housed in standard polycarbonate cages with stainless steel running wheels inside isolation cabinets under light:dark (LD) of 12:12 hours and ambient building temperature 72-78F, (ii) fed 300mg pellets of purified diet (F0075, Bio-Serv) using automated feeders with water provided ad libitum (AL), and (iii) cage changed every 21 days. Nesting material and igloos were excluded, besides shavings, to prevent wheel blockages.

Wild animals: N/A

Reporting on sex: Data for male and female mice are reported separately throughout the manuscript. Female mice exhibited a more prolonged healthspan, whereas lifespan extension was observed only in male mice under 8h-TRF. Female mice also showed a lower incidence of cancer but a higher incidence of renal failure compared to male. With the exception of necropsy data, analyses focused on comparisons between control and time-restricted feeding within each sex, given known sex differences in metabolism, endocrine signaling, and aging. Accordingly, we prioritized within-sex comparisons to evaluate the effects of time-restricted feeding relative to sex-matched controls.

Field-collected samples	N/A
Ethics oversight	This work was approved by The Institutional Animal Care and Use Committee (IACUC) of the University of Texas Southwestern Medical Center under the animal protocol: APN 2015-100925

Note that full information on the approval of the study protocol must also be provided in the manuscript.

Clinical data

Policy information about clinical studies

All manuscripts should comply with the ICMJE guidelines for publication of clinical research and a completed CONSORT checklist must be included with all submissions.

Clinical trial registration	N/A
Study protocol	N/A
Data collection	N/A
Outcomes	N/A

Dual use research of concern

Policy information about dual use research of concern

Hazards

Could the accidental, deliberate or reckless misuse of agents or technologies generated in the work, or the application of information presented in the manuscript, pose a threat to:

No	Yes	
☒	☐	Public health
☒	☐	National security
☒	☐	Crops and/or livestock
☒	☐	Ecosystems
☒	☐	Any other significant area

Experiments of concern

Does the work involve any of these experiments of concern:

No	Yes	
☒	☐	Demonstrate how to render a vaccine ineffective
☒	☐	Confer resistance to therapeutically useful antibiotics or antiviral agents
☒	☐	Enhance the virulence of a pathogen or render a nonpathogen virulent
☒	☐	Increase transmissibility of a pathogen
☒	☐	Alter the host range of a pathogen
☒	☐	Enable evasion of diagnostic/detection modalities
☒	☐	Enable the weaponization of a biological agent or toxin
☒	☐	Any other potentially harmful combination of experiments and agents

Plants

Seed stocks	N/A
Novel plant genotypes	N/A
Authentication	N/A

ChIP-seq

Data deposition

- [ ] Confirm that both raw and final processed data have been deposited in a public database such as GEO.
- [ ] Confirm that you have deposited or provided access to graph files (e.g. BED files) for the called peaks.

Data access links<br>*May remain private before publication.*	N/A
Files in database submission	N/A
Genome browser session<br>(e.g. UCSC)	N/A

Methodology

Replicates	N/A
Sequencing depth	N/A
Antibodies	N/A
Peak calling parameters	N/A
Data quality	N/A
Software	N/A

Flow Cytometry

Plots

Confirm that:

- [ ] The axis labels state the marker and fluorochrome used (e.g. CD4-FITC).
- [ ] The axis scales are clearly visible. Include numbers along axes only for bottom left plot of group (a 'group' is an analysis of identical markers).
- [ ] All plots are contour plots with outliers or pseudocolor plots.
- [ ] A numerical value for number of cells or percentage (with statistics) is provided.

Methodology

Sample preparation	N/A
Instrument	N/A
Software	N/A
Cell population abundance	N/A

Gating strategy: N/A

☐ Tick this box to confirm that a figure exemplifying the gating strategy is provided in the Supplementary Information.

Magnetic resonance imaging

Experimental design

Design type	N/A - Brain imaging not performed, only whole body composition measured every 6 months via EchoMRI. Scans take less than 2 minutes each and require no sedation. Mice are restrained in a tube.
Design specifications	N/A
Behavioral performance measures	N/A

Acquisition

Imaging type(s)	N/A
Field strength	N/A
Sequence & imaging parameters	N/A
Area of acquisition	N/A

Diffusion MRI ☐ Used ☒ Not used

Preprocessing

Preprocessing software	N/A
Normalization	N/A
Normalization template	N/A
Noise and artifact removal	N/A
Volume censoring	N/A

Statistical modeling & inference

Model type and settings	N/A
Effect(s) tested	N/A

Specify type of analysis: ☐ Whole brain ☐ ROI-based ☐ Both

Statistic type for inference (See Eklund et al. 2016)	N/A
Correction	N/A

Models & analysis

n/a	Involved in the study
☒	☐ Functional and/or effective connectivity
☒	☐ Graph analysis
☒	☐ Multivariate modeling or predictive analysis
