# Supplementary material for: Time-restricted feeding extends healthspan in both sexes and lifespan in male C57BL/6 J mice
Source: Nat Aging. 2026 Jun 2;6(6):1227–43. doi: 10.1038/s43587-026-01129-8 (PMC13303084; doi:10.1038/s43587-026-01129-8)
Supplement: Supplementary file 3 — Peer Review File [file 43587_2026_1129_MOESM3_ESM.pdf]

nature portfolio

Peer Review File

Time-Restricted Feeding Extends Healthspan in Both Sexes and Lifespan in Male C57BL/6J Mice

Corresponding Author: Dr Joseph Takahashi

Version 0:

Reviewer comments:

Reviewer #1

(Remarks to the Author)
This study tests the effects of 8-hour and 12-hour time-restricted feeding (TRF) on lifespan and healthspan in male and female C57BL/6J mice. TRF extended healthspan in both sexes (with a greater effect in females) and extended lifespan only in the 8-hour TRF group (with a greater effect in males).

This research has several strengths. The authors used a very large sample size for a murine aging study in order to detect 10% differences in lifespan. This is an extremely difficult and challenging study to pull off, so kudos to the authors. Other strengths include novelty: there are very few studies assessing the effects of TRF on lifespan, and none have investigated the effects in both male and female rodents eating a normal chow diet. The authors also did a very thorough assessment of healthspan, with many endpoints; assessed the cause of death; and performed appropriate statistical tests. Many of the limitations of this mouse model and protocol are adequately addressed in the Discussion. Finally, the study will be of very high interest to readers of Nature Aging and will likely receive considerable public attention.

While there are several issues that need to be addressed, based on the strengths of this study, my recommendation is to accept with minor revisions. most issues can be addressed through textual revisions, clarifications, and additional caveats in the limitations section.

Major Issues:
• There are contradicting statements about the effects of TRF on lifespan in females. The paper states twice that TRF does not extend lifespan in females, yet 8-hour TRF extended median lifespan by 5% as assessed by Fisher's test, even though survival curves were not statistically different. For males, median lifespan is what is reported in the abstract as the primary lifespan endpoint, so it seems that the conclusion should be changed to 8-h TRF does extend median lifespan in females, but the effect is small.
• Animal Strain Selection: The use of the C57BL/6J strain is a primary concern due to its (1) known aberrant circadian phenotypes (depleted melatonin) and (2) inverted sex-based longevity patterns (males outliving females), which complicates the translation of findings into humans. Why were C57BL/6J mice chosen instead of C3H mice if diurnal rhythms were of interest and the authors were not inducing obesity? Please comment on both these points or address this.
• Fasting Duration Calculation: The manuscript reports mean/median fasting duration by averaging the long overnight fast with short inter-pellet pauses during the feeding window. This is misleading and confusing (and also not very informative). Please instead report the *maximum* daily fasting duration instead, which is what defines compliance to the TRF window. Also, convert the units from seconds to hours.
• Feeding Protocol Anomalies: If the mice are restricted to their feeding windows, please address why there are so many red dots indicating food pellet taking during the fasting period for the TRF groups in Figure 1A. This critical anomaly is unexplained and undermines the TRF intervention. It would also be helpful to discuss if the amount of food pellet taking in the AL groups during the inactive period is normal behavior for mice, or if this is an artifact of the pellet release timing, or a strain-specific effect.
• While the writing in the Discussion section is very strong and the writers did a great job anticipating what the reader would want to know, the writing in the other sections needs to be edited in some places for clarity and flow. In particular, the abstract and introduction are somewhat unclear and awkwardly written in some key places. As examples, please reword lines 24-26. The unclear and/or awkward phrases in these lines are ""which self-imposed a caloric restriction" (awkward/unclear), "When integrated into a composite Healthspan Index, these improvements revealed…." (suboptimal grammar), "TRF prolonged healthspan in females more effectively than in males" (unclear whether TRF improved healthspan in males), and "Only 8-hr TRF extended lifespan in males by 12%" (unclear what "only" is modifying, what the effects in females are, and whether the authors are referring to median lifespan or another lifespan metric). Similarly, please work on smoothing out the wording, looking for awkward or unclear phrasing, for instance, by having a fresh set of eyes

review the wording.

Minor Issues:
• Food Hoarding: The methods used to "exclude" data from hoarding animals are unclear, casting doubt on the true fasting durations of these animals. Can more details be provided on how hoarding was measured, and are there any further reassurances that the target fasting durations were achieved? In line 323, how did the authors determine that the mice consumed >90% of food within their TRF window each day if the authors are only collecting hoarded food every 7-21 days? Also please be explicit about how the hoarded food is excluded in the main analyses.
• Circadian Alignment: The feeding window was centered in the middle of the dark (active) phase, which raises questions of whether this is optimally "circadian aligned." It appears that the mid-point of eating for the 8-h groups may have been later than for the 12-h groups. Please add at least one sentence on whether the authors think this affected their results.
• Please comment on whether housing animals at thermoneutrality or without bedding may have influenced (a) the effects of TRF on healthspan and lifespan and (b) any sex-specific differences. The lack of bedding may have influenced the ability of animals to thermoregulate. Please list the ambient temperature and provide the rationale for no bedding. Is it possible that shivering thermogenesis and adipose browning had metabolic effects that modulated the results?
• Missing Data: A Results subsection is titled "TRF...has minimal impacts on glucose homeostasis," yet no glucose homeostasis data are presented in the manuscript.
• Unclear Terminology: In some places, the manuscript fails to clearly distinguish between discussions of animal and human research, potentially causing confusion.
• Please provide the diet composition of the food pellets.
• Figure 5 A and B give the log-rank Mantel-Cox p-values in a way that indicates that test was used for the difference in median lifespan, rather than the Fisher's exact test values that are given in the results section. The 5% increase in median lifespan for females with the significant p-value reported in the results section is not represented in the figure.
• Consider moving the section on "Sex-dependent strategies for achieving a healthy lifespan" from the results to the Supplementary Materials, as it detracts a bit from the overall narrative and reads like a merged "Results and Discussion," as it is more discursive. At a minimum, move some of the discursive elements of this paragraph to the Discussion. Finally, I also have some concerns about the various analyses—such as fat being protective at some ages but not others—to be less convincing or robust, so perhaps some of this should be removed or condensed. Finally, please be specific on the correlation analyses performed here and provide numerical results in the text (not just qualitative statements).
• In line 770, please acknowledge the pros and cons of using a groupwise indication of diurnal amplitude.
• If possible, add clarification on how sex differences in mice may or may not translate to humans.
• It would be helpful to mention that 8-h TRE in mice is not directly translatable to 8-h TRE in humans due to the difference in metabolic rate.
• The mice are not truly ad libitum fed if their food amount is restricted. Please be explicit that the amount of food given is more than the mice can eat in a day and how you arrived at that amount of food, and that no group exceeded the 6.6 g daily consumption.
• What are the implications of the 8-h TRF males being more likely to be underweight (body condition score of 2), and does that affect translatability?
• The mice are all normal weight for their strain early on, but are they not overweight or obese at months 12 and 18? Expected weights: https://www.jax.org/jax-mice-and-services/strain-data-sheet-pages/body-weight-chart-000664
Body condition scores of 4 for the AL group are mentioned, with 3=normal and 5=obese. I am assuming that the body condition score in Figure S8 is the same as mentioned in the text, even though the units are 0.1-0.5 instead of 1-5. Please harmonize this.
• Should some of the data be interpreted as TRE prevented midlife obesity rather than comparing to lifelong healthy, normal-weight subjects?
• Please add a plot of % calorie restriction versus age. This can replace the current plot of food intake in absolute terms, which is less helpful, in my opinion.

Reviewer #2

(Remarks to the Author)
Summary:
This article, authored by Liams, et al., seeks to elucidate the long-term effects of time-restricted feeding (TRF) on the healthspan and lifespan of mice. Most TRF studies on mammals thus far have focused on obese-conditions and glossed over potential sexual dimorphic responses to this dietary intervention. In this paper, the authors took advantage of wheel cages and automated feeders to examine the effect of 12-hour and 8-hour nightly TRF on male and female mice throughout their lives. This long-term experiment showed clear differences in how male and female mice respond to TRF, differences that are enhanced with 8-hour TRF relative to ad lib or 12-hour TRF. While lifespan was only extended significantly in male mice subjected to the 8-hour TRF, healthspan in female mice was extended in both 8-hour and 12-hour when analyzed using a composite healthspan index that takes accounts of both behavioral and body-composition changes. In summary, these experiences demonstrated TRF has sex-specific effects on aging of mice.
Overall, the experiments were well-designed and described; analysis was incredibly thorough; and results were clearly reported and carefully analyzed. Considering the length of time and investment of labor and expense, this manuscript describes a unique effort that will be of significant interest to a broad and general audience. This reviewer especially appreciated the investigation of sex-specific effects of TRF on mammals and the concept of healthspan index. Minor concerns are detailed below; it is difficult to recommend new experiments considering the effort that that would entail. Thus, while this paper does not delve into uncovering the mechanisms that may underlie observed differences between ad lib and TRF or sex-based differences, this reviewer believes that the findings of this paper provide an excellent analytical framework for examining aging-related healthspan and lay important groundwork for future TRF/aging studies.

Minor concerns:
1. The authors do not address cardiac or cardiovascular health. While this reviewer understands that this may not be addressable retrospectively, the authors should state if they have any data or insight on the effects of TRE on cardiac/cardiovascular health. The effect of TRE on cardiac/cardiovascular health has been somewhat controversial in the recent past, with some studies pointing to better health and at least one pointing to much worse health (indirectly). Is there anything in the mouse data (besides fat and weight) that might indicate a position for this study on this topic? I.e., blood pressure or heart arrhythmia? If not, this topic should at least be acknowledged in the discussion.
2. Other TRE studies (from humans to flies) have not been performed for the entire lifespan as this study did. For example, lifespan appears to be extended for Drosophila only when TRE is performed within a specific time window (Catterson et al., Ulgherait et al.). The authors should address the pros and cons of their approach in the context of other more limited, shorter term treatments; it seems possible, particularly given the effects on fat, that TRE is maximally beneficial within a particular window and that TRE might have greater lifespan extension effects if it was ended before old age.
3. The effects on neoplasia are interesting, especially considering the putative links between circadian regulation and cell cycle/growth. While experiments are outside the scope of this paper, this reviewer hopes that this might be a future direction for research. Do the authors have thoughts for the discussion on this topic?
4. In general, all bar graphs should show individual data points or be shown as violin plots or box-or-whisker plots to better reflect variability across the experimental groups. This applies especially to Figure 4F and the Supplementary Figures 2-4.
5. It's curious that the 8-hr TRF mice seem to have significantly higher mean fasting time while the median is lower compared to the ad libitum controls. It would be interesting to take a closer look at how the distribution of the data points contributed to this observation.
6. The result section header on line 112 claims TRF has minimal impacts on glucose homeostasis but it's unclear if the authors showed sufficient data or discussion based on results posted in Figure 3 for this claim.

Reviewer #3

(Remarks to the Author)
The study by Iiams et al. describes the effects of life-long TRF on mice's healthspan, indicating that circadian-aligned fasting enhances various health metrics, behavioral rhythms, metabolic outcomes, and delays disease marker appearance. Interestingly, 12 hours of fasting are sufficient to provide some benefits, while an 8-hour fasting window—which causes self-imposed caloric restriction- provides more significant advantage.
The significance of the article is based on rigorous methodology and data analysis, with salient features including the duration of the intervention, which spans from early adulthood to death; the power of the sample studied; and uncovering sex-specific effects and benefits of fasting, which surprisingly, while extending longevity in males, seems to improve overall health and delay the onset of age-related diseases in females.
The experimental design and statistics are appropriate and support valid conclusions. My main concern is that the study appears observational, and despite the large power provided by the substantial cohort used and the time invested, it falls short in offering mechanistic insights into the molecular pathways through which TRF confers benefits on health and longevity. What about metabolic, energetic, and inflammatory markers? While, in its current version the study has preclinical value in supporting the adoption of less extreme regimens with high potential for translation to healthy humans, it fails to identify the substrate mediating the interaction between fasting, caloric restriction, and longevity in a sex-specific manner.
Other questions/concerns:
1- Animals were housed individually at 2 months of age, apparently without nesting material, in what the authors describe as challenging conditions. A more detailed discussion on how this early environmental change might affect health and lifespan is needed. This paradigm represents a limitation in directly applying findings to human studies.
2- The significance of disease incidence in the pathology analysis must be reported. For example, is the 2-fold increase in the number of male mice with bronchoalveolar carcinoma under TRF a concern?
3- The statement that "higher activity levels and amplitudes consistently correlated with longer lifespan…supports activity as a valuable healthspan biomarker" seems expected. Is the ability to continue exercising a true marker of healthspan, or does prolonged exercise actually promote better health rather than serve as a biomarker, indicating the functioning of the musculo-skeletal and nervous systems?
Fig 5C. Please include R values of correlation and p-values in the scale to better understand the strength of the relationships. Overall, this panel is quite busy, and it may better to find other ways of representing multiple longitudinal relations.
It will be helpful to indicate the developmental or life stage of the animals at each measurement point, rather than just mentioning days. Including key developmental and aging milestones, like hormonal changes in females, will enhance the interpretation.
6- It appears females were never pregnant or used for reproduction (methods are unclear). While avoiding confounders in data analysis, this restricts data interpretation due to missing key hormonal interactions (as discussed).
7- It is unclear whether TRF significantly impacted frailty indexes, as the section mainly examines aging.
Cognitive function declines with age in wild-type mice. Was cognition tested in TRF versus ALF mice? Preserving memory and cognitive abilities during aging is essential as a measure of successful longevity.
8- The rationale for selecting 12h and 8h fasting windows is not explained. How do these durations relate to human behavior? The key question for human research is how much fasting is necessary to gain health benefits; therefore, the potential for applying the 12h fasting approach is high regard to long-term practicality.
References are appropriate and represent well the work in the field, and the mansucrtip is presented with clarity.

Version 1:

Reviewer comments:

Reviewer #1

(Remarks to the Author)
I have carefully reviewed the revised manuscript and the author responses. Overall, the authors have done an excellent and thorough job of responding to all suggestions.

Congratulations to the authors on such important research!

The one minor thing is upon review of this version of the manuscript, I did catch the new statement that "normality was assumed but not formally tested" (line 1114) and the p-values for some of the new supplemental figures were given as "NS", rather than numerical values. It's not necessary in my book to correct this, but if the other reviewers do end up asking the authors to go through a third round of review, then it would be nice to fix this. I don't feel strongly, though.

Reviewer #2

(Remarks to the Author)
The authors have responded to the previous critiques to this reviewer's satisfaction. I have no further comments.

Reviewer #3

(Remarks to the Author)
The authors have made a detailed effort in their revisions and successfully addressed all concerns.
I recommend the manuscript for publication.

Open Access This Peer Review File is licensed under a Creative Commons Attribution 4.0 International License, which permits use, sharing, adaptation, distribution and reproduction in any medium or format, as long as you give appropriate credit to the original author(s) and the source, provide a link to the Creative Commons license, and indicate if changes were made.
In cases where reviewers are anonymous, credit should be given to 'Anonymous Referee' and the source.
The images or other third party material in this Peer Review File are included in the article’s Creative Commons license, unless indicated otherwise in a credit line to the material. If material is not included in the article’s Creative Commons license and your intended use is not permitted by statutory regulation or exceeds the permitted use, you will need to obtain permission directly from the copyright holder.
To view a copy of this license, visit https://creativecommons.org/licenses/by/4.0/

Referee expertise:

Referee #1: TRE, diet

Referee #2: TRE, lifespan

Referee #3: neuro-focus

Reviewers' Comments:
Reviewer #1 (Remarks to the Author):

This study tests the effects of 8-hour and 12-hour time-restricted feeding (TRF) on lifespan and healthspan in male and female C57BL/6J mice. TRF extended healthspan in both sexes (with a greater effect in females) and extended lifespan only in the 8-hour TRF group (with a greater effect in males).

This research has several strengths. The authors used a very large sample size for a murine aging study in order to detect 10% differences in lifespan. This is an extremely difficult and challenging study to pull off, so kudos to the authors. Other strengths include novelty: there are very few studies assessing the effects of TRF on lifespan, and none have investigated the effects in both male and female rodents eating a normal chow diet. The authors also did a very thorough assessment of healthspan, with many endpoints; assessed the cause of death; and performed appropriate statistical tests. Many of the limitations of this mouse model and protocol are adequately addressed in the Discussion. Finally, the study will be of very high interest to readers of Nature Aging and will likely receive considerable public attention.

While there are several issues that need to be addressed, based on the strengths of this study, my recommendation is to accept with minor revisions. most issues can be addressed through textual revisions, clarifications, and additional caveats in the limitations section.
We thank the reviewer for their thoughtful and constructive evaluation of our work. We are grateful for the positive recommendation and for the helpful suggestions to further strengthen the manuscript.
Below, we address each of the reviewer's comments point-by-point, and we have incorporated all requested clarifications, textual revisions, and additional caveats into the revised manuscript. Changes within the manuscript have been highlighted by blue text.

Major Issues:
• There are contradicting statements about the effects of TRF on lifespan in females. The paper states twice that TRF does not extend lifespan in females, yet 8-hour TRF extended median lifespan by 5% as assessed by Fisher's test, even though survival curves were not statistically different. For males, median lifespan is what is reported in the abstract as the primary lifespan endpoint, so it seems that the conclusion should be changed to 8-h TRF does extend median lifespan in females, but the effect is small.
We thank the reviewer for highlighting this inconsistency. Upon re-evaluation, we recognize that the use of Fisher's exact test to assess differences in median lifespan in females was inappropriate. Fisher's exact test collapses time-to-event data into a binary outcome and does not account for censoring or the timing of events. As such, it is not appropriate for assessing differences in median lifespan when survival curves are compared using Kaplan-Meier and log-rank analyses. Fisher's exact test may be applied to maximal lifespan comparisons, where the majority of animals have already experienced the event.

We have therefore removed Fisher's exact test for median lifespan comparison from the manuscript and supplemental tables. Consistent with the log-rank Mantel-Cox analysis, we now conclude that 8h-TRF does not significantly extend lifespan in females. All text, figures, and the abstract have been revised accordingly.

The revised conclusions emphasize that TRF improves healthspan in both sexes, while robust lifespan extension is observed only in males, reflecting a sex-specific effect.

• Animal Strain Selection: The use of the C57BL/6J strain is a primary concern due to its (1) known aberrant circadian phenotypes (depleted melatonin) and (2) inverted sex-based longevity patterns (males outliving females), which complicates the translation of findings into humans. Why were C57BL/6J mice chosen instead of C3H mice if diurnal rhythms were of interest and the authors were not inducing obesity? Please comment on both these points or address this.
We thank the reviewer for highlighting important considerations regarding strain selection. We agree that C57BL/6J mice have well-known limitations, including no melatonin production and their atypical sex-specific longevity pattern, as we observed here. Our rationale for selecting this strain was threefold. First, C57BL/6J remains the most widely used genetic background in circadian and recently aging research, including nearly all prior mouse TRF studies, enabling direct comparison with existing literature and facilitating reproducibility. Second, despite no melatonin, C57BL/6J mice exhibit robust circadian behavioral and metabolic rhythms that respond reliably to feeding schedules, making them suitable for assessing TRF effects. Third, using a

single well-characterized strain allowed us to prioritize statistical power and lifespan resolution in an extremely large cohort.
Conversely, C3H mice, while melatonin-proficient, are highly susceptible to early-onset pathologies such as mammary tumors in females and liver tumors in both sexes, which frequently truncate lifespan and complicate the study of natural aging trajectories. They are also prone to retinal degeneration which makes them blind by adulthood.

As with various C57BL/6J aging studies, our males lived longer than females, which makes direct translational comparisons between sexes challenging. However, by including sex-matched ad libitum-fed controls this enables us to determine the impact of TRF relative to each sex's natural aging trajectory, allowing meaningful interpretation despite the strain's atypical longevity pattern.

- Stehle, J.H., et al. (2002). "Organisation of the circadian system in melatonin-proficient C3H and melatonin-deficient C57BL mice: a comparative investigation. *Cell Tissue Res*, 309, 173–182.
- Banks G., et al. (2015). "Genetic background influences age-related decline in visual and nonvisual retinal responses, circadian rhythms, and sleep. *Neurobiol Aging,* 36(1):380-93.
- Pfeffer, M., et al. (2022). "The Role of the Melatoninergic System in Circadian and Seasonal Rhythms-Insights From Different Mouse Strains." *Frontiers in Physiology*, 13.

We have now included a brief justification of strain selection in the **Methods.**

• Fasting Duration Calculation: The manuscript reports mean/median fasting duration by averaging the long overnight fast with short inter-pellet pauses during the feeding window. This is misleading and confusing (and also not very informative). Please instead report the *maximum* daily fasting duration instead, which is what defines compliance to the TRF window. Also, convert the units from seconds to hours.
We agree that reporting maximum daily fasting duration is more meaningful. Accordingly, we have replaced the median fasting duration plot in Fig. 2D with maximum daily fasting times in hours for each mouse and group. We also replaced the mean fasting figure in Fig. 2C with longitudinal plots showing the time required for each group to take 90% of their daily food intake from the feeder. This is also reported in hours. Together, these analyses suggest that the mice closely adhered to their assigned TRF regimens; for example, the 8h-TRF group took ~90% of their food within 8 h and exhibited an average daily max fasting duration of ~15 h, approximating the intended 8 h feeding/16 h fasting schedule.

These two parameters have also now been incorporated into the ridgeline plots and healthspan index (Fig. 6) and now have helped to show that TRF extends healthspan in both sexes, not just females.

• Feeding Protocol Anomalies: If the mice are restricted to their feeding windows, please address why there are so many red dots indicating food pellet taking during the fasting period for the TRF groups in Figure 1A. This critical anomaly is unexplained and undermines the TRF intervention. It would also be helpful to discuss if the amount of food pellet taking in the AL groups during the inactive period is normal behavior for mice, or if this is an artifact of the pellet release timing, or a strain-specific effect.
Yes, this is a very important point. We have now expanded the **Results** section to include a description of the daytime pellet taking seen in the actograms. As now described in the results, this is due to a technical limitation of the feeder. Once a pellet is dropped it cannot be retracted or blocked, allowing some mice to retrieve the pellet during the light phase. However, accounting for the average daily food intake for each of the TRF groups and sexes across their lifespan, this is <7% of their intake. The majority of the pellets are still consumed within the feeding window. While this is an unintended artifact of the feeder system, we still found significant health and lifespan benefits in the mice. Furthermore, this enhances translatability as most humans are unlikely to be strict or "perfect" throughout a TRE intervention. This latter point we have currently mentioned at the end of the **Discussion.**

"However, a single daytime pellet accounted for ≤6.34% of total caloric intake, and after subtracting hoarded food, mice consumed ≥90% of the pellets taken from the feeder during the correct feeding windows. These findings, along with evidence that TRF 5 out of 7 days per week regimens still improves metabolic health in mice (39), and lifespan in flies, suggest that moderate TRE adherence may still prolong healthspan and be compatible with modern human lifestyles."

For the AL mice, this is normal for C57BL/6J mice to consume a portion of their food during the light phase. We and other studies have previously observed this. We have also now addressed this in the **Results** section.

- Acosta-Rodriguez, V., et al. (2022). "Circadian alignment of early onset caloric restriction promotes longevity in male C57BL/6J mice." *Science* 376(6598): 1192-1202.
- Acosta-Rodriguez, V. A., et al. (2017). "Mice under Caloric Restriction Self-Impose a Temporal Restriction of Food Intake as Revealed by an Automated Feeder System." *Cell Metab* 26(1): 267-277 e262.
- Kohsaka A., et al. (2007). "High-fat diet disrupts behavioral and molecular circadian rhythms in mice." *Cell Metab*. 6:414–421.

- Laposky A.D., et al. (2008). “Sleep and circadian rhythms: key components in the regulation of energy metabolism.” *FEBS Lett*. 582:142–151.

• While the writing in the Discussion section is very strong and the writers did a great job anticipating what the reader would want to know, the writing in the other sections needs to be edited in some places for clarity and flow. In particular, the abstract and introduction are somewhat unclear and awkwardly written in some key places. As examples, please reword lines 24-26. The unclear and/or awkward phrases in these lines are “”which self-imposed a caloric restriction” (awkward/unclear), “When integrated into a composite Healthspan Index, these improvements revealed….” (suboptimal grammar), “TRF prolonged healthspan in females more effectively than in males” (unclear whether TRF improved healthspan in males), and "Only 8-hr TRF extended lifespan in males by 12%" (unclear what "only" is modifying, what the effects in females are, and whether the authors are referring to median lifespan or another lifespan metric). Similarly, please work on smoothing out the wording, looking for awkward or unclear phrasing, for instance, by having a fresh set of eyes review the wording.
Thank you for pointing this out. We have gone through and edited out some of the awkward phrasing as well as had others review the wording throughout the text.

Minor Issues:
• Food Hoarding: The methods used to "exclude" data from hoarding animals are unclear, casting doubt on the true fasting durations of these animals. Can more details be provided on how hoarding was measured, and are there any further reassurances that the target fasting durations were achieved? In line 323, how did the authors determine that the mice consumed >90% of food within their TRF window each day if the authors are only collecting hoarded food every 7-21 days? Also please be explicit about how the hoarded food is excluded in the main analyses.
We thank the reviewer for raising this important point and agree that additional clarification was needed. We have now expanded the **Methods** section to explicitly describe how food hoarding was measured, quantified, and excluded from intake calculations. We have also added a new **Supplementary** plot Fig. S1B and a description in the results to quantitatively show that outside TRF-window pellet taking is <7% (Fig. S1B) of total food retrieval and overall hoarding is less than 10-12% per 21-day period (Fig. S1C).
While we cannot precisely confirm that each mouse consumed >88–90% of their pellets within each daily TRF window, or that each mouse maintained exact fasting durations, the data indicate that the vast majority of food is consumed within the intended feeding times. This is also now further supported by the updated figures in Fig. 2C and 2D for 90% feeding duration and maximum fasting duration. Importantly, this degree of

adherence is sufficient to confer the observed health and lifespan benefits. As noted above, this mirrors what might be expected in humans, who are unlikely to maintain perfectly rigid feeding schedules over a lifetime, a point made in the **Discussion**. Accordingly, we have noted in the **Results** that this suggests most calories are consumed within the feeding window.

• Circadian Alignment: The feeding window was centered in the middle of the dark (active) phase, which raises questions of whether this is optimally "circadian aligned." It appears that the mid-point of eating for the 8-h groups may have been later than for the 12-h groups. Please add at least one sentence on whether the authors think this affected their results.
Another important consideration. When designing this experiment, we decided to place the 8h feeding window within the middle of the night rather than the start of the night as studies have shown that delaying breakfast and having an early dinner under 8h-TRF provides health benefits in humans. This could have an impact on aging outcomes.

- Parr, E.B., et al. (2020). " A Delayed Morning and Earlier Evening Time-Restricted Feeding Protocol for Improving Glycemic Control and Dietary Adherence in Men with Overweight/Obesity: A Randomized Controlled Trial." *Nutrients.* 12(2).

This point has been added to the **Discussion**: “Beyond the life stage at which TRF is initiated, the specific timing of the feeding window relative to the internal circadian clock represents a critical variable in metabolic optimization. In this study, the 8h-TRF window was intentionally positioned in the middle of the active period (ZT14–ZT22), instead of the onset (ZT12), as this is consistent with several previous mouse studies (27, 107), and prior work in humans suggests that delaying breakfast and/or advancing the final meal can confer metabolic benefits. Thus, it remains possible that the enhanced healthspan and lifespan benefits observed with 8h-TRF reflect not only feeding duration, but also a shift in the midpoint of daily caloric intake, an aspect of circadian alignment that warrants further investigation.”

• Please comment on whether housing animals at thermoneutrality or without bedding may have influenced (a) the effects of TRF on healthspan and lifespan and (b) any sex-specific differences. The lack of bedding may have influenced the ability of animals to thermoregulate. Please list the ambient temperature and provide the rationale for no bedding. Is it possible that shivering thermogenesis and adipose browning had metabolic effects that modulated the results?
All mice in this study were housed at an ambient temperature of 72-78°F consistent with standard vivarium conditions, now listed in the **Methods**. The decision to house animals without bedding was driven by technical requirements of the automated feeding and

wheel monitoring system, which necessitates unobstructed access to pellets and accurate detection of feeding and wheel-running behaviors. This has been clarified in the **Methods.** Importantly, this housing condition was applied uniformly across all groups and therefore does not differentially affect comparisons between AL, 12h-TRF, and 8h-TRF animals.

We acknowledge that housing below thermoneutrality and the absence of bedding can increase thermogenic demand, potentially engaging shivering thermogenesis and brown adipose tissue (BAT) activity. This can be addressed in future studies using RNA-Sequencing in BAT. However, because these conditions were constant across all groups, they are unlikely to account for the relative differences observed in healthspan and lifespan to TRF within each sex.

Regarding sex-specific effects, we highlight thermogenesis in the **Discussion** as a major possibility for the unusual shorter lifespan of females. Future studies with nesting material will be important to determine whether this closes in or reverses the lifespan outcomes between the sexes.

For the two points above, the following comment has been added to the **Discussion** “Future studies will be needed to directly evaluate thermogenic pathways underlying these sex-specific effects, including gene expression in brown adipose tissue. In addition, evaluating TRF and lifespan outcomes under thermoneutral housing conditions or with access to nesting material will help disentangle the relative roles of thermoregulation and feeding timing in shaping healthspan and longevity.”

• Missing Data: A Results subsection is titled "TRF...has minimal impacts on glucose homeostasis," yet no glucose homeostasis data are presented in the manuscript.
Thanks for catching this. During editing, we included fasting glucose and glucose tolerance testing results but given the minimal results, we had removed them for this initial submission. We forgot to edit the results title for this.
This data has now been added back into the **Supplemental Figures** and is described in the **Results.** We have also included additional metabolic phenotyping experiments in the **Supplemental Figures** and **Results** including metabolic cage data showing impacts to energy expenditure and respiratory exchange ratio and profiles of metabolic and inflammatory markers from plasma.

• Unclear Terminology: In some places, the manuscript fails to clearly distinguish between discussions of animal and human research, potentially causing confusion.
We have gone through the manuscript and clarified whether references were being made to animal or human studies.

• Please provide the diet composition of the food pellets.
The diet composition and ingredients have been added to the methodology section under "**Animals and Housing Conditions"**

• Figure 5 A and B give the log-rank Mantel-Cox p-values in a way that indicates that test was used for the difference in median lifespan, rather than the Fisher's exact test values that are given in the results section. The 5% increase in median lifespan for females with the significant p-value reported in the results section is not represented in the figure.
As explained above, we determined that Fisher's exact test is not appropriate for assessing differences in median lifespan and have therefore removed all Fisher's exact test-based analyses from the lifespan analyses, with the exception of its use for maximal lifespan comparisons. Accordingly, Figure 5A now reports only log-rank Mantel-Cox p-values, which reflect comparisons of the full survival curves.

• Consider moving the section on "Sex-dependent strategies for achieving a healthy lifespan" from the results to the Supplementary Materials, as it detracts a bit from the overall narrative and reads like a merged "Results and Discussion," as it is more discursive. At a minimum, move some of the discursive elements of this paragraph to the Discussion. Finally, I also have some concerns about the various analyses—such as fat being protective at some ages but not others—to be less convincing or robust, so perhaps some of this should be removed or condensed. Finally, please be specific on the correlation analyses performed here and provide numerical results in the text (not just qualitative statements).
As other reviewers have shared a similar concern, and the data fluctuates in a confusing pattern age-block by age-block, we have decided to remove this figure and section from the manuscript entirely. This figure will be more meaningful in a future publication with more longevity cohorts included and extended analysis.

• In line 770, please acknowledge the pros and cons of using a groupwise indication of diurnal amplitude.
The primary advantage of assessing diurnal amplitude at the group level is the ability to perform inferential statistics, which allows us to discern the impact of our feeding interventions from the inherent individual biological variability. In research, we rarely care about how Mouse X compares to Mouse Y, instead addressing the impact that our experimental manipulations have on a sample of mice.

We acknowledge that this type of analysis leads to a loss of the unique characteristics of behavior that animals display on an individual level. Often these unique behavioral

patterns are neglected as simply noise, however, there is a large amount of information to be gained from assessing the changing behavior of an individual mouse over the course of an experiment, or its lifetime, beyond what can be captured at a group-wise level. For example, young female C57BL/6J mice have a strong pattern of activity that fluctuates on a ~5-day cycle, in line with their estrous cycle. If this mouse somehow lost this rhythmicity, a groupwise averaging of its behavior would completely miss this fact. The truth may be far more interesting, showing that the intervention did indeed lead to a suppression of the estrous cycle.

As it pertains to our study, assessing individual behavioral characteristics is beyond our intended scope, as our primary aim was to assess the robust, reproducible effects of feeding interventions on the population. We use a group-wise assessment of 24h diurnal rhythms as a validated indicator of the overarching impact of each intervention.

• If possible, add clarification on how sex differences in mice may or may not translate to humans.
The concern that sex-differences may not translate directly to humans, especially for duration and magnitude, has been pointed out in the **Discussion** in the section describing the male vs female differences in healthspan and lifespan.

"It is also important to note, that sex differences observed in mice may not fully translate to humans, as differences in circadian rhythms, hormonal cycles and reproduction, and metabolic rate can alter the magnitude and timing of sex-specific responses to TRF."

• It would be helpful to mention that 8-h TRE in mice is not directly translatable to 8-h TRE in humans due to the difference in metabolic rate.
This has now been pointed out in the **Discussion** with references, following the critique on 8h-TRF shifting the midpoint of feeding vs 12h-TRF.

"While we observed robust benefits of 8h-TRF in mice, it is also important to note that direct translation to humans is limited by differences in metabolic rate and circadian timing. In practice, fasting intervals in mice correspond to approximately ~2.7–3.6 times longer relative windows in humans, so caution is warranted when extrapolating the duration and timing of TRF across species."

• The mice are not truly ad libitum fed if their food amount is restricted. Please be explicit that the amount of food given is more than the mice can eat in a day and how you arrived at that amount of food, and that no group exceeded the 6.6 g daily consumption.

We have now updated the **Methods** to clarify our choice and reasoning for choosing 6.6.g of food per day. This exceeds the strain specific characteristic of consuming 4-5g of food per day, ensuring AL mice were not calorically restricted.

However, as seen in Fig. 1B, feeding does increase with age. In the males, they never approach this threshold, suggesting they were sufficiently fed. Females came close but as seen in the figure, females never exceeded 6.15g of food (AL peak) and only showed this level of consumption near the end of life. The point that the mice never reached the maximum 6.6g has been explicitly stated now in the **Results.**

• What are the implications of the 8-h TRF males being more likely to be underweight (body condition score of 2), and does that affect translatability?

This unlikely due to a detrimental effect of 8h-TRF itself, but rather a consequence of this group living substantially longer than both AL and 12h-TRF males. As shown by the average body condition scores at 30 months, and within the **Supplemental Figure** (Fig. S12) for male frailty body condition scores, there is no significant difference between groups. Thus, the higher proportion of low body-condition scores in the 8h-TRF health reports most likely reflects the greater number of mice surviving into very advanced ages, when declines in weight and condition are common across all groups. We feel this does not limit translatability, as the phenomenon reflects extended survival rather than a TRF-specific deficit.

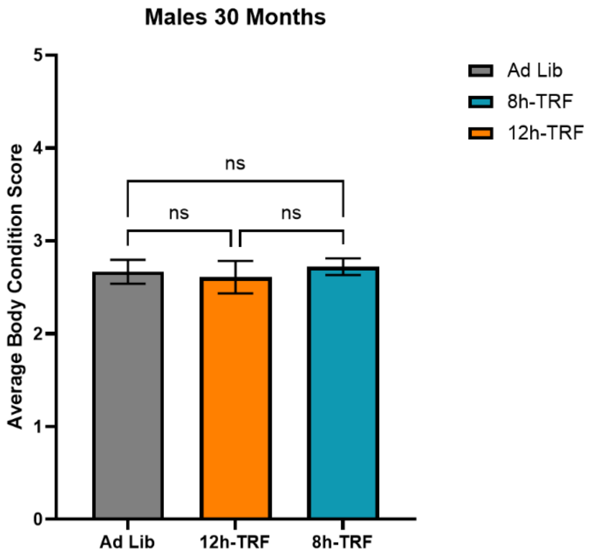

This has now been commented on in the **Discussion**.

“Veterinary health reports were largely sex-dependent, and overall TRF did not alter the types of deficits observed. Notably, 8h-TRF males displayed a higher proportion of low body condition scores (score of 2), but this occurred alongside a delayed onset of health issues and overall increased lifespan and likely reflects the survival of more mice to advanced ages rather than a detrimental effect of TRF.”

• The mice are all normal weight for their strain early on, but are they not overweight or obese at months 12 and 18? Expected weights: https://www.jax.org/jax-mice-and-services/strain-data-sheet-pages/body-weight-chart-000664

For aged mice, our data appear to follow normal trends: https://www.jax.org/jax-mice-and-services/strain-data-sheet-pages/body-weight-chart-aged-b6. At 12 months (48 weeks, males = 44.2 ± 5.3; females = 32.5 ± 4.9) and 18 months (72 weeks), males = 49.7 ± 7.4; females = 37.6 ± 6.2) of age, the AL mice fall within the expected range of body weights.

• Body condition scores of 4 for the AL group are mentioned, with 3=normal and 5=obese. I am assuming that the body condition score in Figure S8 is the same as mentioned in the text, even though the units are 0.1-0.5 instead of 1-5. Please harmonize this.
Mice are scored as 1 (emaciated), 2, 3, 4, or 5 (obese) with 3 representing ideal body condition. For frailty scoring, a mild score of 0.5 was used for any mouse with a body condition of 2 or 4. A severe score of 1 was used for any mouse with a body condition of 1 or 5. This has now been clarified in the **Results** and frailty **Methods.**

• Should some of the data be interpreted as TRE prevented midlife obesity rather than comparing to lifelong healthy, normal-weight subjects?
Obesity is a relative term and not precisely defined in mice. It is generally used to describe animals whose body weight or fat exceeds the expected range for their age and sex, and as shown above, even AL-fed mice in our study fall within these expected ranges. However, we have updated the text to avoid labeling the mice as non-obese, replacing these instances in the **Introduction** with wording that clarifies we did not diet- or genetically-induce obesity. Similarly, we removed references to non-obese mice from the **Discussion**. Importantly, this does not change the conclusion that TRF limited midlife increases in body weight and fat.

• Please add a plot of % calorie restriction versus age. This can replace the current plot of food intake in absolute terms, which is less helpful, in my opinion.
Thank you for this suggestion. We have added plots to Figure 1B that show the % self-imposed calorie restriction relative to AL throughout age. We do prefer to still keep the current food plots as well, as there are very few studies that have measured food intake with hoarding removed continuously throughout the life of C57BL/6J mice. These age-related changes, especially between males and females, may be insightful for other researchers.

Reviewer #2 (Remarks to the Author):

Summary:

This article, authored by Iiams, et al., seeks to elucidate the long-term effects of time-restricted feeding (TRF) on the healthspan and lifespan of mice. Most TRF studies on mammals thus far have focused on obese-conditions and glossed over potential sexual dimorphic responses to this dietary intervention. In this paper, the authors took advantage of wheel cages and automated feeders to examine the effect of 12-hour and 8-hour nightly TRF on male and female mice throughout their lives. This long-term experiment showed clear differences in how male and female mice respond to TRF, differences that are enhanced with 8-hour TRF relative to ad lib or 12-hour TRF. While lifespan was only extended significantly in male mice subjected to the 8-hour TRF, healthspan in female mice was extended in both 8-hour and 12-hour when analyzed using a composite healthspan index that takes accounts of both behavioral and body-composition changes. In summary, these experiences demonstrated TRF has sex-specific effects on aging of mice.
Overall, the experiments were well-designed and described; analysis was incredibly thorough; and results were clearly reported and carefully analyzed. Considering the length of time and investment of labor and expense, this manuscript describes a unique effort that will be of significant interest to a broad and general audience. This reviewer especially appreciated the investigation of sex-specific effects of TRF on mammals and the concept of healthspan index. Minor concerns are detailed below; it is difficult to recommend new experiments considering the effort that that would entail. Thus, while this paper does not delve into uncovering the mechanisms that may underlie observed differences between ad lib and TRF or sex-based differences, this reviewer believes that the findings of this paper provide an excellent analytical framework for examining aging-related healthspan and lay important groundwork for future TRF/aging studies.
We thank the reviewer for their thoughtful and encouraging assessment of our work. We greatly appreciate the recognition of the unique, long-term scope of our study and the careful analysis of sex-specific effects of TRF on healthspan and lifespan. We have addressed the reviewer’s minor concerns and clarifications in the revised manuscript, with changes highlighted in blue text for ease of reference.

Minor concerns:
1. The authors do not address cardiac or cardiovascular health. While this reviewer understands that this may not be addressable retrospectively, the authors should state if they have any data or insight on the effects of TRE on cardiac/cardiovascular health. The effect of TRE on cardiac/cardiovascular health has been somewhat controversial in the recent past, with some studies pointing to better health and at least one pointing to much worse health (indirectly). Is there anything in the mouse data (besides fat and weight) that might indicate a position for this study on this topic? I.e., blood pressure or heart arrhythmia? If not, this topic should at least be acknowledged in the discussion.

We agree that the effects of time-restricted eating on cardiac and cardiovascular health remains an area of active study. We do want to point out though, that the recent study suggesting much worse health does not properly report the cause and age of death in these patients, and has poor records for mealtime adherence, nutrition of the meal, duration of the intervention, and even the health status of the individual before starting the TRF regimen. To avoid stirring up controversy we would like to not introduce this study to the paper. (We are addressing this in a separate review)

However, direct cardiovascular endpoints such as blood pressure, cardiac function, or arrhythmias were not assessed in this study. From the necropsy data, there were mice that developed myocarditis, but the incidence was low and insignificant between the groups. We have now explicitly acknowledged this limited area of analysis in the **Discussion**.

“The absence of direct cardiovascular assessments remains a limitation, particularly as cardiovascular disease is a prevalent age-associated pathology in mice. Given the limited data from long-term studies directly evaluating cardiovascular effects of TRF/TRE, future studies incorporating dedicated measures of cardiac function, structure, and blood pressure will be essential to determine how timed feeding influences cardiovascular health across the lifespan, particularly under conditions of normal metabolic aging.”

2. Other TRE studies (from humans to flies) have not been performed for the entire lifespan as this study did. For example, lifespan appears to be extended for Drosophila only when TRE is performed within a specific time window (Catterson et al., Ulgherait et al.). The authors should address the pros and cons of their approach in the context of other more limited, shorter term treatments; it seems possible, particularly given the effects on fat, that TRE is maximally beneficial within a particular window and that TRE might have greater lifespan extension effects if it was ended before old age.
This is a great point and has now been added to the **Discussion** following the section discussing the potential of testing this intervention at different starting ages:

“Furthermore, as most of the observed benefits occurred through mid-life and the onset of old age, this raises the question of whether a lifelong intervention is necessary, or if stopping TRF before old age might be sufficient, or even preferable, to avoid potential late-life trade-offs. This possibility is supported by other models, where short-term TRF applied in flies earlier in life increases longevity and functional health even after returning to AL feeding, and by some evidence that TRF in aged mice had negative impacts on lean mass and cardiovascular health.”

3. The effects on neoplasia are interesting, especially considering the putative links between circadian regulation and cell cycle/growth. While experiments are outside the scope of this paper, this reviewer hopes that this might be a future direction for research. Do the authors have thoughts for the discussion on this topic?
We thank the reviewer for highlighting this point. We agree that the links between circadian regulation and cell cycle control are highly relevant to the observed sex-specific differences in neoplasia. While mechanistic experiments are beyond the scope of the current study, we have added a statement to the **Discussion**.

"Although mechanistic experiments are beyond the scope of this study, RNA-sequencing and proteomic analysis of the liver, the tissue most affected by neoplasia in males and least in females, could help to dissect the molecular mechanisms underlying these sex-specific differences in cancer development under TRF. These mechanisms are likely to include pathways regulating the cell cycle and cellular proliferation."

4. In general, all bar graphs should show individual data points or be shown as violin plots or box-or-whisker plots to better reflect variability across the experimental groups. This applies especially to Figure 4F and the Supplementary Figures 2-4.
Thank you for pointing this out. All bar graphs now have the individual data points displayed or were converted to dot plots or box plots as visually appropriate.

5. It's curious that the 8-hr TRF mice seem to have significantly higher mean fasting time while the median is lower compared to the ad libitum controls. It would be interesting to take a closer look at how the distribution of the data points contributed to this observation.
As other reviewers have pointed out, the median fasting plot is a bit confusing and misleading. We have instead replaced this plot in Fig. 2D to show maximum fasting throughout life. We have also replaced the mean fasting figure in Fig. 2C with longitudinal plots of the time it takes each group to take 90% of their daily food intake. Together these two plots better show that the mice have strong adherence to their TRF regimens. We hope that the conclusion is now more meaningful and strengthens the results of the paper.

We have now integrated these two parameters into the ridgeline plots and healthspan index (Fig. 6), which clarifies that TRF promotes healthspan extension in both sexes, not exclusively in females.

6. The result section header on line 112 claims TRF has minimal impacts on glucose

homeostasis but it's unclear if the authors showed sufficient data or discussion based on results posted in Figure 3 for this claim.
This was a mistake on our part during editing. Fasting glucose and glucose tolerance testing results had minimal impacts, so we thought to exclude it on the initial submission, only to forget to remove it from the result title.

However, this data has now been added back into the **Supplemental Figures** and is described in the **Results.** We have also included additional metabolic phenotyping experiments in the **Supplemental Figures** and **Results** including metabolic cage data showing impacts to energy expenditure and respiratory exchange ratio, and profiles of metabolic and inflammatory markers from plasma.

Reviewer #3 (Remarks to the Author):

The study by Iiams et al. describes the effects of life-long TRF on mice's healthspan, indicating that circadian-aligned fasting enhances various health metrics, behavioral rhythms, metabolic outcomes, and delays disease marker appearance.
Interestingly, 12 hours of fasting are sufficient to provide some benefits, while an 8-hour fasting window—which causes self-imposed caloric restriction- provides more significant advantage.
The significance of the article is based on rigorous methodology and data analysis, with salient features including the duration of the intervention, which spans from early adulthood to death; the power of the sample studied; and uncovering sex-specific effects and benefits of fasting, which surprisingly, while extending longevity in males, seems to improve overall health and delay the onset of age-related diseases in females.
The experimental design and statistics are appropriate and support valid conclusions.
My main concern is that the study appears observational, and despite the large power provided by the substantial cohort used and the time invested, it falls short in offering mechanistic insights into the molecular pathways through which TRF confers benefits on health and longevity. What about metabolic, energetic, and inflammatory markers?
While, in its current version the study has preclinical value in supporting the adoption of less extreme regimens with high potential for translation to healthy humans, it fails to identify the substrate mediating the interaction between fasting, caloric restriction, and longevity in a sex-specific manner.
We thank the reviewer for their thoughtful evaluation and positive comments regarding the rigor, scope, and translational value of our study. We have addressed the points raised regarding metabolic, energetic, and inflammatory markers, as well as sex-specific health outcomes, and these additions are highlighted in blue text throughout the

manuscript. While mechanistic experiments remain beyond the scope of this work, the newly included data provide more physiological markers and insight into the safety of TRF, including longitudinal assessments of energy expenditure and respiratory exchange ratio, glucose tolerance, circulating metabolic and inflammatory markers, and hematological health. Surprisingly, we found minimal or no impacts on these markers, suggesting in a non-obesity inducing state other metabolic markers are playing a role in the health and aging benefits observed here.

Other questions/concerns:
1- Animals were housed individually at 2 months of age, apparently without nesting material, in what the authors describe as challenging conditions. A more detailed discussion on how this early environmental change might affect health and lifespan is needed. This paradigm represents a limitation in directly applying findings to human studies.
This is an important point and the potential impact of individual housing without nesting material is discussed as a limitation in the **Discussion**:
"While genetic factors may play a role, the shorter lifespan in females could stem from enhanced cold sensitivity and higher thermoregulatory energy demands. The smaller bodied females, needing more energy to maintain body temperature as evidenced by the pronounced increase in food intake with age, may have been adversely affected by the harsher individual housing conditions without nesting material. Although lower body temperatures can extend lifespan, cold stress can also divert energy away from and impair metabolic, immune, and reproductive functions."

We have now added additional discussion following this proposing future studies under thermoneutral housing conditions or with access to nesting material to disentangle the effects of thermoregulation from feeding timing on healthspan and longevity.

"Future studies will be needed to directly evaluate thermogenic pathways underlying these sex-specific effects, including gene expression in brown adipose tissue. In addition, evaluating TRF and lifespan outcomes under thermoneutral housing conditions or with access to nesting material will help disentangle the relative roles of thermoregulation and feeding timing in shaping healthspan and longevity."

2- The significance of disease incidence in the pathology analysis must be reported. For example, is the 2-fold increase in the number of male mice with bronchoalveolar carcinoma under TRF a concern?
We agree that reporting the statistical significance of disease incidence is essential and have now explicitly included these analyses. Fisher's exact tests were performed for all disease categories shown in Fig. 5B, and the corresponding P values are provided in

the newly added Table S7. In the revised **Results**, we now specify which neoplastic subtypes were significantly altered by TRF and include the associated statistical comparisons.
While bronchioloalveolar adenomas were significantly increased in both 12h-TRF and 8h-TRF males relative to AL controls ($P < 0.05$), the incidence of bronchoalveolar carcinoma was not significantly different between feeding conditions (Table S7). Thus, the apparent increase reflects a rise in benign lung tumors rather than malignant disease. Consistent with this, overall disease burden was not exacerbated under TRF, and TRF males exhibited delayed onset of health decline. This is also commented on now in the **Results.**

3- The statement that "higher activity levels and amplitudes consistently correlated with longer lifespan…supports activity as a valuable healthspan biomarker" seems expected. Is the ability to continue exercising a true marker of healthspan, or does prolonged exercise actually promote better health rather than serve as a biomarker, indicating the functioning of the musculo-skeletal and nervous systems?
Please see following answer.

Fig 5C. Please include R values of correlation and p-values in the scale to better understand the strength of the relationships. Overall, this panel is quite busy, and it may better to find other ways of representing multiple longitudinal relations.
It will be helpful to indicate the developmental or life stage of the animals at each measurement point, rather than just mentioning days. Including key developmental and aging milestones, like hormonal changes in females, will enhance the interpretation.
Addressing both of these above points. As you and other reviewers have pointed out, this figure is very busy but also rather difficult to interpret. To avoid adding confusing results and conclusions regarding these correlations, we have decided to remove this individual health marker vs lifespan correlation figure. This analysis will be more useful in a future publication when more aging cohorts can be included and more extensive analysis performed.

6- It appears females were never pregnant or used for reproduction (methods are unclear). While avoiding confounders in data analysis, this restricts data interpretation due to missing key hormonal interactions (as discussed).
We have now clarified in the **Methods** that all of the mice are unmated before being placed into individual cages. This point has also now been highlighted in the **Discussion** that translation may be limited due to differences in hormonal cycles and reproduction.

“It is also important to note, that sex differences observed in mice may not fully translate to humans, as differences in circadian rhythms, hormonal cycles and reproduction, and metabolic rate can alter the magnitude and timing of sex-specific responses to TRF.”

7- It is unclear whether TRF significantly impacted frailty indexes, as the section mainly examines aging.

Sorry this was unclear. As shown in Figure 4A and **Supplemental Figures** S11–S12, TRF significantly reduced frailty index scores at specific ages in both sexes, with 12h-TRF lowering frailty index at 18 months of age in females and at 12 and 18 months of age in males. 8h-TRF had significantly reduced frailty indices compared to AL at 12, 18 and 24 months of age. The effects were dose- and sex-dependent, with 8h-TRF providing the most prolonged improvements and impacting the largest number of individual health measures. We have clarified this in the **Results.**

Cognitive function declines with age in wild-type mice. Was cognition tested in TRF versus ALF mice? Preserving memory and cognitive abilities during aging is essential as a measure of successful longevity.

Cognition was not directly tested in this study. Although, from our metabolic and inflammatory panel, we did test circulating levels of BDNF and found no impact on levels by TRF with exception of 8h-TRF males at 6 months of age. This may reflect a brief neuroprotective benefit, but this is lost at later ages. This result can now be found in the **Results** in the “**Circulating metabolic and inflammatory markers”** section.

8- The rationale for selecting 12h and 8h fasting windows is not explained. How do these durations relate to human behavior? The key question for human research is how much fasting is necessary to gain health benefits; therefore, the potential for applying the 12h fasting approach is high regard to long-term practicality.

Thank you for highlighting this. The 12h and 8h feeding windows were selected based on previously validated TRF regimens in mice, which reliably produce metabolic and circadian benefits. This is now clarified in the **Methods**.

We also note that due to species differences in metabolic rate and circadian timing, these windows do not directly translate to humans; fasting windows in humans correspond to almost a 2.7–3.6-fold shorter window than in mice. This is now pointed out as a limitation and commented on in the **Discussion.**

“While we observed robust benefits of 8h-TRF in mice, it is also important to note that direct translation to humans is limited by differences in metabolic rate and circadian timing. In practice, fasting intervals in mice correspond to approximately ~2.7–3.6 times longer relative windows in humans, so caution is warranted when extrapolating the duration and timing of TRF across species.”

References are appropriate and represent well the work in the field, and the manuscript is presented with clarity.
